# Identifying the true number of specimens of the extinct blue antelope (*Hippotragus leucophaeus*)

**DOI:** 10.1038/s41598-020-80142-2

**Published:** 2021-01-22

**Authors:** Elisabeth Hempel, Faysal Bibi, J. Tyler Faith, James S. Brink, Daniela C. Kalthoff, Pepijn Kamminga, Johanna L. A. Paijmans, Michael V. Westbury, Michael Hofreiter, Frank E. Zachos

**Affiliations:** 1 Evolutionary Adaptive Genomics, Universität Potsdam, Karl-Liebknecht-Str. 24-25, 14476 Potsdam, Germany; 2 Museum Für Naturkunde, Leibniz Institute for Evolution and Biodiversity Science, Invalidenstraße 43, 10115 Berlin, Germany; 3 Natural History Museum of Utah, University of Utah, 301 Wakara Way, Salt Lake City, UT 84108 USA; 4 Department of Anthropology, University of Utah, 260 South Central Campus Drive, Salt Lake City, UT 84112 USA; 5 Florisbad Quaternary Research Station and Department, National Museum Bloemfontein, P.O. Box 266, Bloemfontein, 9031 Republic of South Africa; 6 Centre for Environmental Management, University of the Free State, PO Box 339, Bloemfontein, 9300 Republic of South Africa; 7 Swedish Museum of Natural History, Box 50007, 10405 Stockholm, Sweden; 8 Naturalis Biodiversity Center, Darwinweg 2, 2333 CR Leiden, The Netherlands; 9 Section for Evolutionary Genomics, The GLOBE Institute, University of Copenhagen, Øster Voldgade 5-7, Copenhagen, Denmark; 10 Natural History Museum Vienna, Burgring 7, 1010 Vienna, Austria; 11 Department of Evolutionary Biology, University of Vienna, Vienna, Austria; 12 Department of Genetics, University of the Free State, Bloemfontein, South Africa; 13 Present Address: Department of Genetics and Genome Biology, University of Leicester, Leicester, LE1 7RH UK

**Keywords:** Evolution, Genetics, Zoology

## Abstract

Native to southern Africa, the blue antelope (*Hippotragus leucophaeus*) is the only large African mammal species known to have become extinct in historical times. However, it was poorly documented prior to its extinction ~ 1800 AD, and many of the small number of museum specimens attributed to it are taxonomically contentious. This places limitations on our understanding of its morphology, ecology, and the mechanisms responsible for its demise. We retrieved genetic information from ten of the sixteen putative blue antelope museum specimens using both shotgun sequencing and mitochondrial genome target capture in an attempt to resolve the uncertainty surrounding the identification of these specimens. We found that only four of the ten investigated specimens, and not a single skull, represent the blue antelope. This indicates that the true number of historical museum specimens of the blue antelope is even smaller than previously thought, and therefore hardly any reference material is available for morphometric, comparative and genetic studies. Our study highlights how genetics can be used to identify rare species in natural history collections where other methods may fail or when records are scarce. Additionally, we present an improved mitochondrial reference genome for the blue antelope as well as one complete and two partial mitochondrial genomes. A first analysis of these mitochondrial genomes indicates low levels of maternal genetic diversity in the ‘museum population’, possibly confirming previous results that blue antelope population size was already low at the time of the European colonization of South Africa.

## Introduction

**Figure 1:**
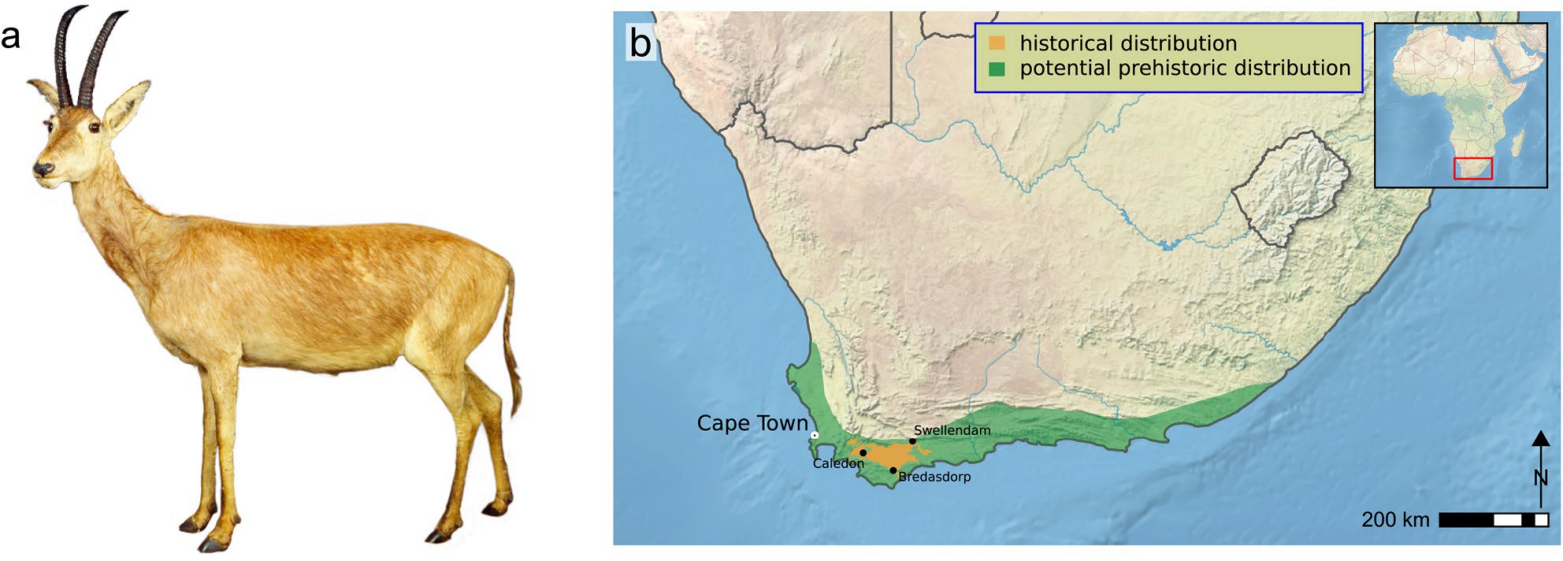
The blue antelope and its historical and potential prehistoric distribution. (**a**) The only known mounted female specimen of the blue antelope (*Hippotragus leucophaeus*) (Natural History Museum Vienna, NMW ST 715); the characteristic white patch in front of the eyes is clearly visible (photo credit: Natural History Museum Vienna). (**b**) Historical and potential prehistoric distribution of the blue antelope (drawn in Inkscape v0.91 (https://inkscape.org) from Kerley et al.^16^ and Faith & Thompson^6^; base map: https://www.naturalearthdata.com v2.0.0).

The blue antelope, *Hippotragus leucophaeus* (Pallas, 1766), is the first and only large African mammal species to become extinct in historical times^1,2^ (Fig. 1a). Endemic to southern Africa, during the seventeenth and eighteenth century, the blue antelope (also called bluebuck, blaauwbok or bloubok) was confined to a very limited range between Caledon, Swellendam and Bredasdorp^3,4^ (Fig. 1b). However, the Pleistocene and Holocene fossil record documents its presence across a much larger, albeit still relatively restricted, area along the coastal plains of the Cape Floristic Region^5–7^ and possibly also in the highlands of Lesotho^8^. Its name derives from its pelt color, which was perceived to be bluish-grey while the animal was alive, with a distinctive white patch in front of the eye (*leucophaeus* is Greek for ‘white–grey’)^9,10^ (Fig. 1a).

The blue antelope was likely first mentioned in writing by J. Schreyer in 1679^4,11^. P. Kolb^12^ was the first to characterize it in more detail when he encountered it during his travels near Caledon in 1708. It was first formally described as *Antilope leucophaea* in 1766 by P. S. Pallas, based on several skins in The Hague^9^. Around 1800, only about 100 years after it was first mentioned, the blue antelope went extinct^13,14^.

Its extinction has generally been attributed to competition with and habitat deterioration by livestock^5,15^, disruption of migratory pathways both in prehistoric times and through Colonial-era habitat transformation^6^, and overhunting by European colonists^1,2^. Using a modelling approach, Kerley et al.^16^ found that its population size was already small (about 370 individuals) with a small distribution range, and that the species therefore was highly vulnerable at the time of European colonization (mid-seventeenth century). These authors concluded that hunting by European colonists drove the blue antelope to extinction, but that it was not the only force leading to its demise. The growing demand of European collectors to own a specimen of this already declining species (e.g. Rookmaaker)^4^ might have contributed as well^16^. Low genetic diversity in historical samples would provide further support for low population sizes in blue antelope, though this has not been investigated yet. In fact, the knowledge of the blue antelope’s evolutionary history and ecology is very limited and originates from just a few early and, as was common at the time, anecdotal travel reports, the archaeological and paleontological records, and deductions from observations of its extant congeners, the roan (*H*. *equinus*) and the sable (*H*. *niger*), both of which were described later (in 1803 and 1838, respectively).

The early extinction of the blue antelope is reflected in its scarcity in natural history collections today. Additionally, almost all specimens purported to be of the blue antelope lack secure provenance and are of uncertain taxonomic identity. For more detailed information on provenance and about the specimens in general see Supplementary Document S1. Mohr^17^, Rookmaaker^18^ and Renshaw^19^ collated lists of existing, lost, and potential specimens. To the best of our knowledge, only 16 specimens are recorded as potential blue antelopes in museums (15, if the skull fragment and the mounted skin from Leiden are from the same individual; see "Samples" section). All but one of these are housed in Europe. These comprise four mounted skins (Leiden, Paris, Stockholm, Vienna), six skulls or crania (Berlin, Glasgow, Leiden, Paris), one skull fragment (in Leiden, possibly originating from the mounted skin there), four pairs of horns (Cape Town, London, St Andrews, Uppsala), and one specimen that is either a skull or a pair of horns (in Brussels, currently not accessible due to renovation). Given the morphological similarities with roan and sable antelope and the lack of confirmed reference material, the taxonomic identity of many of these specimens, particularly the skulls, is contentious (e.g. Mohr^17^, Groves & Westwood)^20^. This raises the important question: how many of them can truly be assigned to the blue antelope? Several morphological studies have attempted to determine the species identity of these specimens^17,20,21^, but with unsatisfactory results. Two studies have successfully extracted blue antelope DNA from museum specimens, each working with only a single specimen: Robinson et al.^22^ analyzed 502 bp of the *CYTB* gene from the Vienna specimen, and Themudo & Campos^23^ generated a mitochondrial genome from the Uppsala specimen.

In the present study, we analyzed ten potential blue antelope specimens. We generated a revised complete mitochondrial genome, performed phylogenetic analyses and, in the tradition of Mohr^17^, Rookmaaker^18^ and Renshaw^19^, present a revised list of true blue antelope museum specimens. The genetically confirmed taxonomic identity of these specimens will facilitate morphological research on the blue antelope and reduce the present taxonomic uncertainty. We also present a first preliminary estimate of the genetic diversity of the blue antelope ’museum population’ by comparing two complete and two partial blue antelope mitochondrial genomes.

## Material and methods

### Samples

A thorough museum record and literature search for specimens currently or previously labeled as blue antelopes resulted in a list of 16 potential historical blue antelope specimens (e.g. Mohr^17^, Rookmaaker^18^, Husson & Holthuis^24^, Erdbrink)^25^: four mounted skins, six skulls/crania, one skull fragment, four pairs of horns, some with frontlet, and one inaccessible specimen that is either a skull or a pair of horns (Table 1, Supplementary Document S1).

**Table 1:** List of definitive, potential and previously assumed historical blue antelope specimens.

ID	Museum	Specimen type	Sex	Sample material (this study)	Species identification	Accession numbers
NRM 590107	Swedish Museum of Natural History (Naturhistoriska riksmuseet, Stockholm)	Mounted skin	Male	Skin	*H*. *leucophaeus*	MW222233
NMW ST 715	Natural History Museum Vienna	Mounted skin	Female	Skin	*H. leucophaeus*	MW228401
RMNH.MAM.20681.a (Leiden 1)	Naturalis Biodiversity Center (Leiden)	Premaxilla and mandible fragments	Male(?)	Bone	*H. leucophaeus*	MW228402
UPSZMC 78488	Museum of Evolution (University of Uppsala)	Pair of horns with traces of fur	Unknown	Skin and bone	*H. leucophaeus*	MW222234
BPM 2234^a^	Bell Pettigrew Museum (University of St Andrews)	Frontlet with horns	Unknown	Bone	*H. equinus*	MW228403
MNHN-ZM-AC-1896-100^a^	Muséum national d’Histoire naturelle (Paris)	Cranium with horns, no mandible	Unknown	Tooth root	*H. equinus*	MW228404
ZMB MAM 8859	Museum für Naturkunde, Mammal Collection (Berlin)	Cranium with horns, no mandible	Unknown	Bone	*H. equinus (Mohr*^17^ assigned it to *H. equinus* based on morphology)	MW228405
GLAHM:Z4884	The Hunterian (University of Glasgow)	Cranium with horns and mandible, no horn sheaths	Unknown	Bone	*H. niger*	MW228406
SAM ZM 40759	Iziko Museums of South Africa, Terrestrial Vertebrate Collection (Cape Town)	Frontlet with horns	Male(?)	Bone	*H. niger*	MW228407
ZMA.MAM.18623 (Leiden 2)	Naturalis Biodiversity Center (Leiden)	Cranium with horns and mandible	Catalogue: female; Erdbrink^25^: male; Groves and Westwood^20^: female	Bone	*H. niger*	MW228408
RMNH.MAM.20681.b (lectotype)	Naturalis Biodiversity Center (Leiden)	Mounted skin	Male	Not analyzed	*H*. *leucophaeus*	–
MNHN-ZM-MO-1994-1103	Muséum national d’Histoire naturelle (Paris)	Mounted skin	Male	Not analyzed	*H*. *leucophaeus*	–
NHMUK GERM 636e	Natural History Museum London	Frontlet with horns	Unknown	Not analyzed	*H*. *leucophaeus*	–
RBINS 3785^a^	Royal Belgian Institute of Natural Sciences (Brussels)	Skull or horns	Unknown	Not analyzed	*H*. *leucophaeus*	–
ZMB MAM 8855	Museum für Naturkunde, Mammal Collection (Berlin)	Cranium with horns, no mandible	Female	Not analyzed	(Mohr^17^ assigned it to *H*. *niger* based on morphology)	–
ZMB MAM 8860^a^	Museum für Naturkunde, Mammal Collection (Berlin)	Cranium with horns and mandible	Male	Not analyzed	(currently labeled as *H*. *equinus*)	–

The historical specimens which, at some point in time, have been identified as blue antelope (*Hippotragus leucophaeus*), are listed with current species identification. Horn sheaths are present unless stated otherwise. Sex given as in museum catalogue or literature.

^a^Specimens previously not mentioned in the literature.

All institutions housing specimens were contacted and subsequently ten bone and/or skin samples were obtained. Photographs (if available, Supplementary Figs. S1–S15) and detailed information about the specimens are presented in Supplementary Document S1. Whenever documents have been inspected by one of the authors (for example copies of catalogues), no source is stated; whenever the information was provided by others, this is stated as personal communication.

#### Specimens analyzed

The specimen from the Swedish Museum of Natural History (Naturhistoriska riksmuseet) in Stockholm (NRM 590107) is a mounted skin of a subadult male, its young age being obvious from its short horns with few rings. The collector A. U. Grill received the specimen from C. P. Thunberg^26^. In 1829, Grill’s heirs donated it to the Swedish Museum of Natural History^17,18^. It is highly likely that no skull is inside the skin (see Supplementary Document S1). The mounted skin from the Natural History Museum Vienna (NMW ST 715) is the only known female. Recent X-ray analysis (June 2017) has shown that the Vienna specimen does not contain a skull (Supplementary Fig. S2). According to the acquisition catalogue, it became part of the collection in 1806. It was the first specimen of the species from which DNA was successfully recovered^22^.

The complete skull (without horn sheaths) from the Hunterian at the University of Glasgow (GLAHM:Z4884) was discovered there in 1949^21^, whereas the complete skull (including both horn sheaths) in the collection of Naturalis Biodiversity Center in Leiden (ZMA.MAM.18623) was formerly stored in the Zoölogisch Museum in Amsterdam before this collection was merged with Naturalis in 2011. To our knowledge, the cranium at the Muséum national d’Histoire naturelle in Paris (MNHN-ZM-AC-1896-100) has not been previously mentioned in the literature. According to the catalogue, it is from Senegal and was donated by a certain Houdelot(?). It was first mentioned in a museum catalogue at the end of the nineteenth century (pers. comm. Joséphine Lésur, in charge of the Collections Ostéologiques d'Anatomie Comparée at the Muséum national d’Histoire naturelle). Two crania and one skull in the Mammal Collection at the Museum für Naturkunde in Berlin were at some point identified as blue antelopes. Only one of these, a cranium (ZMB MAM 8859) was sampled. It is now cataloged as a roan, possibly after Mohr’s^17^ revision. Its date of arrival in the collection, geographic origin, and sex are unknown.

The skull fragments at Naturalis Biodiversity Center in Leiden consist of the anterior part of a mandible and premaxillary bones (RMNH.MAM.20681.a). They may derive from the same specimen represented also by the mounted skin in this collection (RMNH.MAM.20681.b)^24^ (see Specimens not analyzed). The collection catalogue from 1887 refers to this as a complete skull^24,27^, and it may be that the remainder of the skull is within the mounted skin. Therefore, the fragments bear the same object ID as the mounted skin in the collection catalogue, but with ‘b’ for the mounted skin and ‘a’ for the skull fragments. We refer to the skull fragments as Leiden 1 in this study, to distinguish them from the complete skull from Leiden, which is referred to as Leiden 2.

The horns in the Museum of Evolution at the University of Uppsala (UPSZMC 78488) still have traces of fur attached to their bases, but are not connected by a frontlet. From this specimen, we received a fur/skin as well as a bone sample. It had already been used earlier to generate a mitochondrial genome of the blue antelope^23^. According to Rookmaaker^4^ it was received in 1781 by C. P. Thunberg from D. F. Immelmann.

The frontlet with horns from the Terrestrial Vertebrate Collection at the Iziko Museums of South Africa in Cape Town (SAM ZM 40759) was first reported by Ozinsky^28^. The only available information is that it was part of the private collection of Mr. J. Piek from Observatory (Cape Town) and entered the collection in 1989 (pers. comm. Denise Hamerton, curator Terrestrial Vertebrate Collection at Iziko Museums of South Africa). The frontlet with horns from the Bell Pettigrew Museum at the University of St Andrews (BPM 2234) has, to our knowledge, never been mentioned in the literature. It is labeled as ‘Blaubok? *Ozanna leucophaeus* (Pall.) South Africa, now extinct’. It seems to lack documentation of its history as well as information on its origin.

#### Specimens not analyzed

The mounted skin at Leiden Naturalis Biodiversity Center (formerly Rijksmuseum van Natuurlijke Historie) (RMNH.MAM.20681.b) is an adult male and the lectotype of the species, to which later Swellendam was assigned as location^24^. The mounted skin in the Collections d'Anatomie Comparée at the Muséum national d’Histoire naturelle in Paris is an adult male (MNHN-ZM-MO-1994-1103). It was first mentioned in a museum catalogue from the end of the nineteenth century (pers. comm. Joséphine Lésur; for a recent publication see Glenn)^29^. Mohr^17^ assumed that the Paris specimen did not contain its skull, on account of the shape of its forehead (where the nasal and frontal bones would be). This was confirmed by Péquignot^30^. While the Leiden and Paris mounted skins are most likely true blue antelopes (based on their morphological similarity with the genetically confirmed Stockholm and Vienna specimens), despite the comment in Skead^31^ that a blue antelope could be manufactured ‘using the skin of an animal resembling it’, they should be genetically analyzed in the future.

The Museum für Naturkunde in Berlin houses two more specimens at one time labeled as blue antelopes: the cranium of a female from South Africa (ZMB MAM 8855) and the skull of a male from Sudan (ZMB MAM 8860). Mohr^17^ determined ZMB MAM 8855 to be a sable after receiving measurements from Berlin, which is probably the reason for its relabeling. ZMB MAM 8860 has to our knowledge never been mentioned in the literature; unfortunately, it was discovered too late to be included in our study.

Little information is available for the frontlet with horns in the Natural History Museum in London (NHMUK GERM 636e). In Lydekker’s^32^ catalogue of the ungulate mammals in the British Museum it is listed as ‘provisionally referred to this species [blue antelope]’. It was mentioned by Sclater & Thomas^10^ as having ‘been long in the Museum’. The specimen in the Royal Belgian Institute of Natural Science in Brussels (RBINS 3785) has to our knowledge never been mentioned in the literature and could not be accessed for this study due to collection renovation. According to the museum catalogue, it is either a skull or a pair of horns which was donated to the museum in 1931 by the arachnologist and ichthyologist Dr L. Giltay.

More detailed information about these specimens, as well as others documented in the literature but believed to not exist anymore, is given in Supplementary Document S1 and Supplementary Table S1.

### Laboratory procedures

#### DNA preparation

DNA was extracted from bone using the Dabney et al.^33^ protocol and from skin by using a combination of Rohland et al.^34^ and Dabney et al.^33^ protocols with digestion buffer modifications from Taron et al.^35^ (5 M GuSCN, 25 nM NaCl, 50 mM Tris, 20 mM EDTA, 1% Tween-20, 1% 2-Mercaptoethanol) using columns of Roche’s High Pure Viral Nucleic Acid Kit for purification. DNA extracts were built into single-stranded libraries following Gansauge et al.^36^ with the adjustment of an initial 15 min. incubation step with 0.5 µl USER (Uracil-Specific Excision Reagent) Enzyme for Uracil removal (modified from Meyer et al.)^37^. A maximum of 13 ng DNA was used as input for library construction. qPCR was performed to determine the optimal number of amplification cycles for the subsequently performed dual-indexing PCR. Extraction and library blanks were run alongside all samples to check for contamination. For the sample from Uppsala, a library was built from the bone as well as the skin sample. As both the Stockholm and Vienna samples contained very short reads after initial test-sequencing, both were processed on the Pippin Prep (Sage Science) with a 3% Agarose Gel Cassette for targets from 90–250 bp (Marker C) (product no. CSD3010) with selection parameters set to 165–300 bp to remove short reads and thereby improve the mappable fraction of sequenced reads. All pre-PCR lab work was conducted in dedicated archival DNA facilities at the University of Potsdam, Germany. The libraries were shotgun sequenced using custom primers^38,39^ on an Illumina NextSeq500 at the University of Potsdam generating 75 bp single-end reads.

#### Mitochondrial genome enrichment

For array capture following Hodges et al.^40^ and Paijmans et al.^41^, an Agilent Sure select Array was designed based on the mitochondrial genomes of *Hippotragus leucophaeus* (NC_035309^23^), *H. equinus* (NC_020712)^42^ and *H. niger* (NC_020713)^42^. As *H*. *leucophaeus* contained two areas in the control region, between coordinates 15,643–15,673 and 16,396–16,422, with missing/ambiguous data, these were removed from the bait design. For *H*. *niger*, one ambiguous base within *CYTB* was adjusted to match the corresponding position in *H*. *leucophaeus* and *H*. *equinus* (R, position 14,921). All genomes were divided into 60-mer probes with 3 bp tiling using a custom python script. To avoid underrepresentation of the region where the mitochondrial genome was cut to linearize it, 59 bp from the beginning of each of the mitochondrial genomes were copied to its end. On the same array, mitochondrial genomes for five Caprinae species were present for enrichment of samples from a different project. Prior to capture, libraries were pooled in equimolar amounts according to their mitochondrial endogenous content as determined via shotgun sequencing (Supplementary Table S3 and S4)^43^. Due to its already high endogenous content, the Stockholm sample was not captured. The capture product was sequenced using custom primers on an Illumina NextSeq500 at the University of Potsdam generating 75 bp single-end reads.

### Bioinformatic procedures and analyses

#### Mitochondrial genome reconstruction

For the reconstruction of the complete mitochondrial genome of the blue antelope, both the Stockholm (NRM 590107) and the Vienna (NMW ST 715) specimens were considered since they are both mounted skins and as such their attribution to the blue antelope is likely correct. However, the Stockholm specimen was chosen as it yielded the best quality DNA (highest endogenous content). For all reads, Illumina adapter sequences (1 bp overlap) and reads < 30 bp were removed with Cutadapt v1.12^44^. Next, potential PCR-duplicates were removed by sequence identity (--by-seq) with SeqKit v0.12.0 rmdup^45^ and quality trimming was conducted for a quality of 30 at the 3′ end of the reads with Cutadapt v1.12^44^. For mitochondrial genome assembly, MITObim v1.9.1^46,47^, which employs the mapper Mira v4.0.2^48^, was used with the --quick option and default settings except for the mismatch value and a limit of 500 iterations. All runs stopped before 500 iterations were reached. Details of the run results are shown in Supplementary Table S6. As reference bait sequences, the complete mitochondrial genomes of the two closest relatives *H*. *niger* (NC_020713)^42^ and *H*. *equinus* (NC_020712)^42^ were used. For each bait, mismatch values from zero to six were tested (Supplementary Table S6). The assembled reads were filtered for a minimum mapping quality of 30, sorted using samtools view and sort v0.1.19^49^ and read groups were adjusted using Picard AddOrReplaceReadGroups v2.22.0 (Picard Toolkit 2020—http://broadinstitute.github.io/picard/) before duplicates were removed with samtools rmdup v0.1.19^49^ based on the 5′ mapping coordinate. A consensus sequence was generated for each run using a 75% majority rule threshold for base calling and a minimum coverage of 3x. The consensus sequences were edited such that all started with tRNA-Phe and ended with the control region. The resulting mitochondrial genome differed depending on whether *H*. *niger* or *H*. *equinus* was used in that 91 bp were only present in the control region when *H*. *niger* was used. This could be a bait sequence bias, since there is a region of 78 bp present in the *H*. *niger*, but not in the *H*. *equinus* reference. Therefore, it could not be determined if this region is present in *H*. *leucophaeus* using only these two bait sequences. To infer this, we ran MITObim v1.9.1^46,47^ with single genes, *ND4* and *CYTB*, of *H*. *equinus*, *H*. *niger* and *Capra hircus* (KR059146)^50^ as bait sequences, using the same settings as described before and mismatch values from one to six. The results were treated in the same way as stated above. The complete mitochondrial genome of *H*. *leucophaeus* was then generated by aligning the twelve sequences created using *H*. *niger* and *H*. *equinus* as bait sequences, each with mismatch values from one to six applying the MAFFT algorithm v7.450^51,52^ with default settings as implemented in Geneious R10 v10.2.3^53^ (https://www.geneious.com). A consensus sequence was generated from this alignment, again employing a 75% majority rule threshold for base calling except for the described area in the control region where only the sequences that used *H*. *niger* as bait reference were considered (matching positions 15,676–15,766 in the final sequence). The newly created complete mitochondrial genome of *H*. *leucophaeus* was annotated in Geneious R10 v10.2.3^53^ using the available annotated *H*. *niger* mitochondrial genome (NC_020713)^42^.

#### Read mapping

Data from different sequencing runs were combined per sample before processing. Cutadapt v1.12^44^ was used to trim Illumina adapter sequences (1 bp overlap) and to discard reads shorter than 30 bp. The resulting reads were mapped to the newly constructed *Hippotragus leucophaeus* mitochondrial genome and the two available mitochondrial genomes of *H*. *niger* and *H*. *equinus* from GenBank with the BWA aln algorithm v0.7.8 with default settings^54^. Quality filtering was performed using samtools view v0.1.19^49^ applying a minimum mapping quality filter of 30 and a subsequent sorting step with samtools sort v0.1.19^49^. Duplicates were marked taking both 5′ and 3′ mapping coordinates into account with MarkDupsByStartEnd v0.2.1 (https://github.com/dariober/Java-cafe/tree/master/MarkDupsByStartEnd) and removed with samtools view v0.1.19^49^.

#### Species identification

The reads of each individual were mapped to the complete mitochondrial genomes of the three *Hippotragus* species *H. leucophaeus* (this study), *H. niger* (NC_020713)^42^ and *H. equinus* (NC_020712)^42^ to determine the species identity for each individual. The relative mapping success for each sample was normalized by defining the result of the reference it mapped best to as 1 and dividing the results of each of the other two references by the results of the reference it mapped best to. For each individual, for the reference it mapped best to, a consensus sequence was generated in Geneious R10 v10.2.3^53^ using a 75% majority rule threshold for base calling, a minimum coverage of 3 × and the ‘trim to reference sequence’ option. Positions where an insertion was called relative to the reference were removed if the insertion was covered by less than three reads. To improve the coverage at the edges of the sequences it was necessary to map the obtained reads again to modified versions of the same references. For this, the circular character of the mitochondrial genome was used by cutting the last 100 bp of each reference and concatenating them to the beginning of the sequence to allow reads to map in the region where the reference had been cut before. Consensus sequences were called and insertions removed as described above. Subsequently, the first 100 bp of each sequence were cut and concatenated back to the end of the sequences to match the original reference again. Then the consensus sequences of both mappings were combined using a 50% majority rule threshold for base calling. In a final step, the sequences were edited such that all started with tRNA-Phe and ended with the control region. This resulted in improved sequences for the samples from Glasgow (GLAHM:Z4884), Leiden 1 (RMNH.MAM.20681.a), Berlin (ZMB MAM 8859), Paris (MNHN-ZM-AC-1896-100), St Andrews (BPM 2234), Stockholm (NRM 590107), Uppsala (UPSZMC 78488) and Vienna (NMW ST 715).

An alignment applying the MAFFT algorithm v7.450^51,52^ to the consensus sequences of the reference that each individual mapped the most base pairs to was built using default parameters as implemented in Geneious R10 v10.2.3^53^ with the gap opening penalty altered to 1.605. In this alignment, the references of *H*. *niger* and *H*. *equinus* from GenBank were included. Since the new reference of *H*. *leucophaeus* is identical to the sample from Stockholm, the reference was not included. As outgroup the mouflon (*Ovis aries musimon*) was included (HM236184)^55^. The control region from each sequence was removed from the alignment because this region is challenging to align for divergent sequences. The final alignment (four taxa) was used to generate a maximum-likelihood tree with 100 bootstrap replicates using RAxML 8.2.10 with the GTR + G substitution model (Fig. 3)^56^.

To rule out the influence of reference bias, a consensus sequence for each sample for each of the three *Hippotragus* references was generated in Geneious R10 v10.2.3^53^, again using a 75% majority rule threshold for base calling, a minimum coverage of 3 × and the ‘trim to reference sequence’ option. No insertions were deleted and no improvement of the edges of the sequences was performed. The control region was removed. An alignment and a maximum-likelihood tree using RAxML 8.2.10 with GTR + G^56^ were built separately for the consensus sequences of each of the three references using the parameters described above (Supplementary Figs. S16–S18). The alignments were improved by shifting gaps by hand.

#### Intraspecific mitochondrial diversity comparison

The consensus sequences of the four specimens identified as blue antelopes (see "Results" section) were aligned using the MAFFT algorithm v7.450^51,52^ with default parameters as implemented in Geneious R10 v10.2.3^53^. All ambiguities including missing data were removed from the alignment, leaving 6,300 bp. Subsequently, from this alignment a median joining network was built and the nucleotide diversity calculated using POPART v1.7^57^ with default parameters.

#### Interspecific mitochondrial diversity comparison

The genetic diversity of the blue antelope specimens was estimated with an average pairwise distance (k) approach, where k is the average number of substitutions in a pairwise individual comparison. For comparison, six wild ungulate species with a wide range of characteristics were chosen: one closely related species (scimitar-horned oryx, *Oryx dammah*), three species that are known to have low genetic diversity, including the European bison (*Bison bonasus*), the American bison (*Bison bison*) and the Przewalski’s horse (*Equus przewalskii*), as well as two species with large geographic ranges, the African buffalo (*Syncerus caffer*) and the moose (*Alces alces*). For each of the six comparison species, all sequences considered reliable were downloaded from GenBank. If a sequence was uploaded to GenBank only once, but was found in more than one individual within a study, it was included as many times as individuals with it were present in the respective study (Supplementary Table S5). The control region of each sequence was removed. All sequences were aligned together with the four blue antelope consensus sequences (also without the control region) using the MAFFT algorithm v7.450^51,52^ with default settings as implemented in Geneious R10 v10.2.3^53^. All positions containing ambiguities/missing data or gaps were excluded from the alignment to ensure that only homologous positions were compared. Subsequently, for each species four sequences were randomly drawn without replacement using seqtk v1.3 (https://github.com/lh3/seqtk), then aligned with MAFFT v7.453^51,52^ using the default settings for gap opening penalty (--op 1.53) and offset value (--ep 0.123). The pairwise distance was calculated using the command line version of MEGA-CC 10^58,59^ applying the ‘complete deletion’ option for the treatment of gaps and missing data. This was repeated 1,000 times for each of the species. Finally, the average was calculated using an R script. For the blue antelope, this was performed once with the four available sequences. The results were visualized using RStudio v1.1.423^60^ (https://www.rstudio.com/) with R v3.63^61^ (https://www.R-project.org/).

## Results

Processing the Stockholm and Vienna samples on the Pippin Prep resulted in 4.5 × more mappable endogenous data for the Stockholm and 13.5 × more for the Vienna sample compared to their respective original libraries. For the mitochondrial genome reconstruction, the results for mismatch values zero contained many unknown bases. Therefore, this value appeared too strict and the results were dismissed. The results using *H*. *niger* as assembly bait sequence for mismatch values one and two were identical (both A at position 15,784, C at position 15,786) as well as for mismatch values three to six (R at position 15,784, Y at position 15,786). The same was true when using *H*. *equinus* (values 1 & 2: A at position 15,769 and C at position 15,770; values 3–6: R at position 15,769 and Y at position 15,770). One area of 91 bp in the control region was present when using *H*. *niger*, but not when using *H*. *equinus* as bait sequence. This might be a bait sequence bias since in this area a region of 78 bp is present in *H*. *niger*, but not in the *H*. *equinus* reference. No run with a single gene as assembly bait sequence resulted in a complete mitochondrial genome. However, the area in question was covered when using *CYTB* with mismatch values one to six and *ND4* with mismatch values four to six, irrespective of the species used as a bait sequence. The results for the area in question were in each case identical to the one obtained when using the complete *H*. *niger* sequence as bait.

The complete mitochondrial genome of *Hippotragus leucophaeus* (Stockholm specimen) is 16,514 bp long and consists of 13 protein coding genes, 22 tRNAs, 2 rRNAs and the control region as expected for a mammalian mitochondrial genome.

For each sample all reads from all runs were combined resulting in 4,751,815—26,107,111 75 bp single end reads (Supplementary Table S2).

### Species identity of the museum specimens

**Figure 2:**
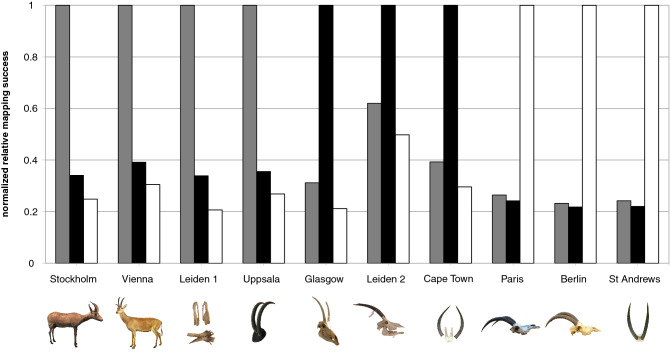
Mapping success to the three *Hippotragus* references. Comparison of normalized relative mapping success for each sample as base pairs mapping to the mitochondrial genomes of the three *Hippotragus* species. For each sample the result of the reference it mapped best to was defined as a mapping success of 1 and the results for the other references are given in relation to that. Only four specimens can unambiguously be assigned to the blue antelope (grey), while three are identified as sable (black) and three as roan (white). Photo credits: Glasgow: Photo: E. Hempel, courtesy: the Hunterian, University of Glasgow; Berlin: Photo: E. Hempel, courtesy: Museum für Naturkunde, Berlin, Mammal Collection; Cape Town: Photo: J. T. Faith, courtesy: Terrestrial Vertebrate Collection, Iziko Museums of South Africa; Leiden 1 & 2: Naturalis Biodiversity Center, the Netherlands; St Andrews: University of St Andrews; Stockholm: Swedish Museum of Natural History; Vienna: Natural History Museum Vienna; Paris: Muséum national d’Histoire naturelle, Collections d'Anatomie Comparée; Uppsala: Museum of Evolution.

The mitochondrial mapping success rates show that the mounted skins from Stockholm (NRM 590107) and Vienna (NMW ST 715), the skull fragments from Leiden 1 (RMNH.MAM.20681.a) and the pair of horns from Uppsala (UPSZMC 78488) are conspecific. Since Leiden 1 is probably part of the skull of the blue antelope lectotype specimen and the Stockholm and Vienna mounts are blue antelopes beyond reasonable doubt based on their morphology, these four specimens can be attributed to *Hippotragus leucophaeus* (Fig. 2, Supplementary Table S2).

**Figure 3:**
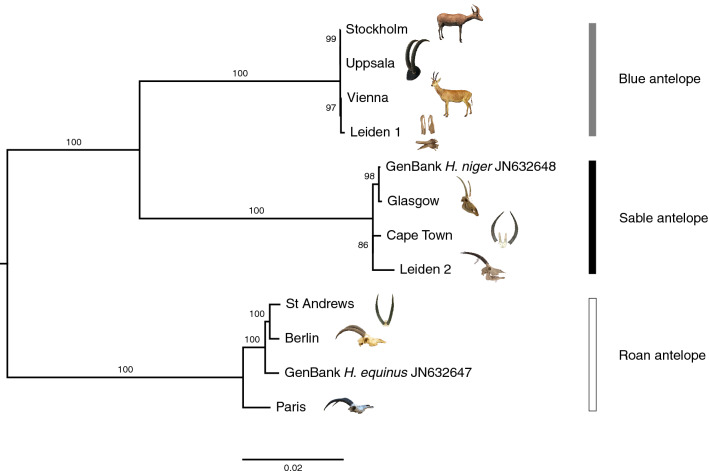
Clustering of samples in a maximum-likelihood phylogeny. Maximum-likelihood tree based on the mitochondrial genome alignment excluding the control region (15,450 bp in length) using the reference with the highest mapping success for each sample. The tree shows three clades (100% bootstrap support each) in agreement with the three *Hippotragus* species (black: sable, white: roan, grey: blue antelope), confirming the species assignments from the mapping results. RAxML 8.2.10 with GTR + G^56^ was used to generate the phylogeny. Branch values indicate bootstrap support of 100 replicates. *Ovis aries musimon* (HM236184)^55^ was used as outgroup (not shown here). Photo credits: see Fig. 2.

The specimens from Paris (MNHN-ZM-AC-1896-100, skull), Berlin (ZMB MAM 8859) and St Andrews (BPM 2234), however, belong to roan (*H. equinus*), while those from Glasgow (GLAHM:Z4884), Leiden 2 (ZMA.MAM.18623) and Cape Town (SAM ZM 40759) are sable (*H. niger*) (Fig. 3, Table 1).

All four blue antelope samples map second best to the sable and all sable specimens map second best to the blue antelope mitochondrial genome. On the other hand, all roan samples map almost equally well to the sable and blue antelope mitochondrial genomes, with a slightly higher value for the blue antelope (Fig. 2).

Complete mitochondrial genomes were generated for Stockholm (104x) and Uppsala (17.7x), and partial mitochondrial genomes were generated for Leiden 1 (3.9x), Vienna (6.2x), Berlin (5.1x), Paris (7.3x), St Andrews (13.7x), Cape Town (2.2x), Glasgow (4.1x) and Leiden 2 (1.7x) (Supplementary Table S7).

The maximum-likelihood phylogeny groups Stockholm (NRM 590107), Vienna (NMW ST 715), Leiden 1 (RMNH.MAM.20681.a) and Uppsala (UPSZMC 78488) together as blue antelopes; Glasgow (GLAHM:Z4884), Leiden 2 (ZMA.MAM.18623) and Cape Town (SAM ZM 40759) with the sable reference; and Paris (MNHN-ZM-AC-1896-100), Berlin (ZMB MAM 8859) and St Andrews (BPM 2234) with the roan reference (Fig. 3). All three clades have 100% bootstrap support. The possibility of a reference bias could be excluded as each of the three maximum-likelihood trees using the same mapping reference for each specimen confirms the above relationships (Supplementary Fig. S16-18).

Since the identity of all tested samples could be clearly established, this allowed for a tentative estimate of the genetic diversity of the blue antelope museum specimens.

### Low levels of genetic diversity in blue antelope museum specimens

**Figure 4:**
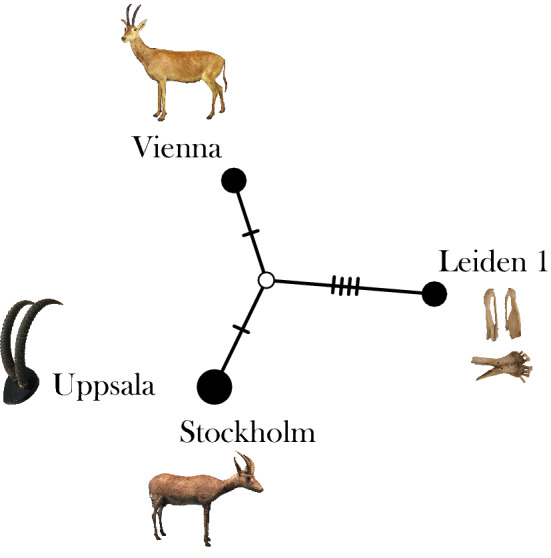
Median joining network of the four blue antelope specimens. The median joining network takes into account only positions of the complete mitochondrial genome (including control region) that are covered in all four specimens, excluding ambiguities (6,300 bp). Dashes and the white circle denote mutational steps and an unsampled haplotype, respectively. The network generated in POPART v1.7^57^ revealed six segregating sites, with the Stockholm and Uppsala specimens being identical in this comparison. Photo credits: Leiden 1: Naturalis Biodiversity Center, the Netherlands; Stockholm: Swedish Museum of Natural History; Vienna: Natural History Museum Vienna; Uppsala: Museum of Evolution.

The comparison of the four unambiguous blue antelope specimens using a median joining network, taking only the positions into account where all specimens had unambiguous available data and including the control region, revealed only six segregating sites (Fig. 4) and a nucleotide diversity (pi) of 0.0005. The sequences of the Stockholm and Uppsala specimens were identical in this comparison.

**Figure 5:**
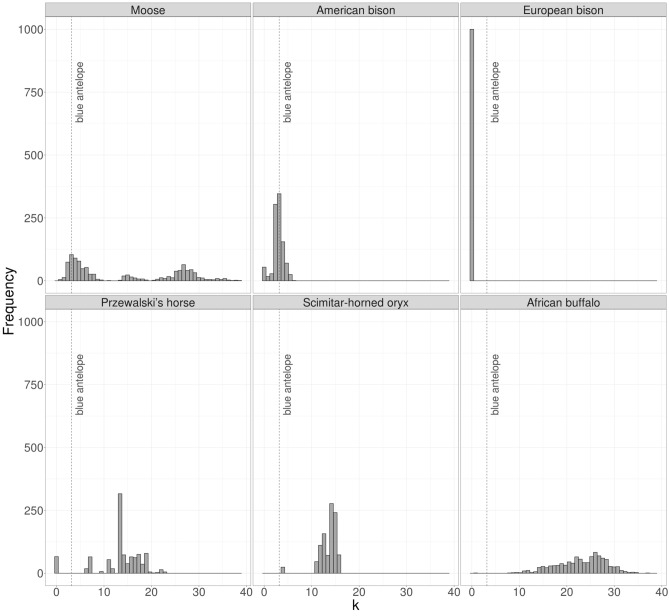
Mitochondrial diversity comparison. Mitochondrial diversity of the four blue antelope specimens (dashed line) compared to the distribution of 1,000 replicates of the diversity of four randomly drawn sequences of six wild ungulate species based on the alignment of homologous positions of the mitochondrial genome (6,061 bp in length) (shown with bins of 50, generated in RStudio v1.1.423^60^ (https://www.rstudio.com/) using R v3.63^61^ (https://www.R-project.org/)). k represents the average number of substitutions between two individuals.

In the pairwise distance comparison, the blue antelope sequences from Stockholm, Vienna, Uppsala and Leiden 1 were compared. The resulting k value was compared to the k values of 1,000 comparisons of four randomly chosen sequences of homologous positions of six wild ungulate species (Fig. 5). In comparison to the other species, the blue antelope shows a rather modest k value (3.2), similar to that in the American bison.

## Discussion

Our results show that only four of the ten analyzed specimens are in fact blue antelopes, while the remaining six belong to either roan or sable. The mapping success per reference species (Fig. 2) together with the well-resolved phylogeny (Fig. 3) yield unequivocal evidence as to the species identity of each analyzed specimen. The pattern that all sable map second best to the blue antelope mitochondrial genome and all blue antelopes map second best to the sable mitochondrial genome (Fig. 2) is in accordance with our phylogeny (Fig. 3) and previous results, indicating that the blue and sable antelope are sister species to the exclusion of the roan^23^.

Our results suggest that historical specimens of the blue antelope in museum collections are even rarer than previously thought (although fossil remains are more abundant, see below). Further work should examine the six remaining potential blue antelope specimens not analyzed here, although two of these are currently listed as sable (ZMB MAM 8855) and roan (ZMB MAM 8860), respectively. The blue antelope is therefore among the rarest historically extinct large mammal species in museums. By comparison, the other recently extinct South African ungulate, the quagga (*Equus quagga quagga*), a subspecies of the plains zebra, is represented by ~ 34 individual specimens^62,63^, and of Steller’s sea cow (*Hydrodamalis gigas*), which became extinct roughly at the same time as the blue antelope, there are at least 27 skeletons, 62 additional skulls and more than 550 bones^64^.

Since our study only analyzed mitochondrial DNA, we cannot exclude the possibility that some of our sampled specimens are hybrids. The roan had overlapping distributions with the blue antelope since at least the Last Glacial Maximum^5,65^, and potentially longer. In historical times Du Plessis^66^ found no evidence of roan antelope as far south as the Cape Floristic Region, but Mohr^17^ considered a sketch by R. J. Gordon from 1778 near Plettenberg Bay to be more likely of a roan than a blue antelope. This specimen is considered to be the holotype of the roan by Grubb^67^. This would make this the last roan to be documented in the southern Cape before its extirpation in that region (yet undescribed as a species)^17,65^. However, population level nuclear DNA data from the blue antelope, which are not currently available, and from species it might have hybridized with, would be required to investigate whether any of the blue antelope museum specimens represent hybrids.

Osteological material, especially in the form of skulls and teeth, is extraordinarily important for comparative morphological, morphometric and taxonomic studies. It is widely used as reference material for museum specimens and archaeological and paleontological material to study and delineate species. For the blue antelope Groves & Westwood^20^ stated that ‘The authentication of even one skull of this extinct antelope would be a significant event.’ With the only historical osteological remains of the blue antelope represented by the skull fragments in Leiden, the horns in Uppsala, and some phalanges within the Vienna mounted skin, our results suggest that there is currently not a single skull that could be used as a reference against which to evaluate historical or fossil specimens. This creates the problem whereby archaeological and paleontological specimens (mostly teeth) identified as blue antelope through morphological characters (namely distinguished from roan by smaller molars and premolars, and from sable by larger premolars)^5,65^ cannot be directly compared with the historical skins, horns and bone fragments identified as blue antelope through DNA analyses. This difficulty is further demonstrated by the many misidentified skulls and frontlets presented in our study, which is a known issue in museum collections^68^. This means that, while the fossil and historical specimens assigned to the blue antelope are very likely one and the same species, this cannot be directly demonstrated. Non-invasive imaging technologies like X-ray or µCT should be applied to investigate whether the Leiden mounted skin possesses a complete (or partial) skull. Ultimately, if a skull is present, it could be scanned, and a cast could be produced by means of a 3D printer. The same could be done for the Stockholm specimen to be certain that it really does not contain a skull.

With only four to maximally ten blue antelope specimens existing, they should be highly valued in museum collections. Since misidentification can go both ways, it is also possible that further specimens of the blue antelope exist, but are mislabeled as sable or roan. This is especially true in light of the fact that their status as separate species was not settled for quite some time^10,69^ and that due to its early extinction, the blue antelope was never well known. Further blue antelope specimens might be privately owned in South Africa or elsewhere. For now, the genetic analysis of known potential blue antelope specimens in collections should be prioritized to perhaps increase the number of unequivocal individuals of this iconic extinct mammal species.

Our sample size is too small to draw well-founded conclusions about the genetic diversity of the blue antelope population in the seventeenth and eighteenth centuries. However, our results suggest that the blue antelope exhibited low genetic diversity in historical times, as previously suspected^16,22^, similar to the low levels seen in the European^70^ and American bison^71^. Low genetic diversity itself does not necessarily lead to extinction^72^, but it can render species more susceptible to diseases^73–76^. However, there is no evidence that the blue antelope was affected in this way. Rather, low genetic diversity itself is likely to be an indication of low population size, and this would certainly have made the blue antelope much more susceptible to extinction in the face of hunting or environmental disturbance, as suggested by Kerley et al.^16^ and Faith & Thompson^6^.

Of the four confirmed blue antelope specimens, all lack verified, detailed location information. Hence, we cannot draw conclusions concerning their geographic origin. However, the two specimens from Swedish museums—Stockholm and Uppsala—have most likely identical mitochondrial genomes, suggesting that they might have originated from the same herd. This seems likely considering that both were acquired by C. P. Thunberg, who was in charge of the collection in Uppsala at the time.

For a better estimate of the genetic diversity of the blue antelope in the seventeenth and eighteenth centuries, larger sample sizes would be needed. For now, this is not feasible since there is an insufficient number of potential blue antelope specimens that remain to be tested. However, there are large numbers of archaeological and paleontological specimens attributed to this species from late Pleistocene and Holocene sites in the southern and western Cape region^7^, with which ancient DNA analyses can be carried out to address questions of past population size changes.

There is an interesting parallel to the bontebok (*Damaliscus pygargus pygargus*) that had a similar historical distribution and was also endemic to the Cape Floristic Region^15,66,77^. Like the blue antelope, the bontebok is thought to have been severely impacted by hunting and pastoralism, resulting in fragmented populations, bringing the species close to extinction at the beginning of the nineteenth century^1,78^. Its genetic diversity is considered to be low today^79^, and its survival is only thanks to the efforts of private landowners who enclosed some of the remaining animals^1^. Unfortunately, the blue antelope went extinct before similar measures could be taken.

The blue antelope was likely one of the inhabitants of the Palaeo-Agulhas plain, the coastal platform off the southern Cape that was exposed during marine regressions (glacials) and provided a nutrient-rich ecosystem that supported diverse ungulate communities. However, during high sea levels (interglacials), the inhabitants would have been restricted to the nutrient-poor and therefore suboptimal fynbos habitats^80–84^ where they might have undergone population fragmentation^6,84^. On the other hand, the blue antelope (like other species) clearly survived such Pleistocene climatic fluctuations without going extinct^6,84,85^. Kerley et al.^16^, based on historical sightings, estimated a total population size of as few as 370 individuals in only a single population at the time of the arrival of the European colonists, which would have rendered the species already highly threatened^6,84,85^. Therefore, it seems likely that other factors must have contributed to its demise in historical times. Resource competition with livestock, habitat deterioration in addition to a suboptimal and probably fragmented habitat likely put the remaining blue antelope population under extreme pressure at the time of European colonization^6,84^. Unsustainable hunting and further habitat transformation at the time would have been the last contributing piece that led to the extinction of the species^84^.

## Conclusions

We show that the blue antelope is not only blue but also lonesome, with only four unequivocal historical specimens in collections today: Stockholm and Vienna (mounted skins), Leiden (skull fragments) and Uppsala (pair of horns). The mounted skins from Leiden and Paris (although the former is probably from the same individual as the skull fragments) can be considered blue antelopes as well. In addition, there are four more potential specimens (in Berlin, Brussels and London). This makes it one of the scarcest historical mammal species in museums, with currently no skull identified that could serve as a comparative morphological reference. Our study is yet another example that misidentifications can be an issue in museum collections, which may seriously impact morphological studies. It highlights the promising potential of the application of archival DNA to resolve existing uncertainties of museum specimens. Furthermore, our preliminary assessment hints at a rather low level of mitochondrial diversity within the blue antelope ‘museum population’.

## Supplementary information

Supplementary Data 1.

Supplementary Data 2.

Supplementary Data 3.

Supplementary Data 4.

Supplementary Data 5.

Supplementary Data 6.

Supplementary Information

## Data Availability

All supplementary material (Supplementary Information/Document S1 and Supplementary Data 1–6) including alignments can be downloaded from the journal’s website at 10.1038/s41598-020-80142-2. All complete and partial mitochondrial genomes were uploaded to GenBank under the accession numbers MW222233-MW222234 and MW228401-MW228408.
