# Supplementary material for: Identifying the true number of specimens of the extinct blue antelope (Hippotragus leucophaeus)
Source: Sci Rep. 2021 Jan 22;11:2100. doi: 10.1038/s41598-020-80142-2 (PMC7822880; doi:10.1038/s41598-020-80142-2)
Supplement: Supplementary file 7 — Supplementary Information [file 41598_2020_80142_MOESM7_ESM.pdf]

Identifying the true number of specimens of the extinct blue antelope (*Hippotragus leucophaeus*)

Elisabeth Hempel, Faysal Bibi, J. Tyler Faith, James S. Brink, Daniela C. Kalthoff, Pepijn Kamminga, Johanna L. A. Paijmans, Michael V. Westbury, Michael Hofreiter and Frank E. Zachos

Supplementary Document S1

Whenever documents have been inspected by one of the authors (for example copies of catalogues), no source is stated; whenever the information was provided by others, this is stated as personal communication.

Information about analyzed specimens - Blue antelopes (*Hippotragus leucophaeus*)

Swedish Museum of Natural History - NRM 590107

The Swedish Museum of Natural History (Naturhistoriska riksmuseet) in Stockholm houses the mounted skin of a young male (NRM 590107) (**Supplementary Fig. S1a**). It was denoted a female in Sundevall's catalogue (~1840), but some years later he called it a juvenile male, without changing this in the catalogue[1] (**Supplementary Fig. S1b**). The blue antelope specimen was first in the collection of Adolf Ulric Grill, who received it from Carl Peter Thunberg[2] and was donated by the heirs of Adolf Ulric Grill to the Swedish Museum of Natural History in 1829. According to Sundevall's catalogue from ~1840, the skull resided inside the specimen ('cr fix') (**Supplementary Fig. S1b**). Mohr[1] reported the museum's intention to remove the skull from the specimen, though this appears to not have happened. A recent investigation of the mounted skin suggests that no bone, not even a frontlet between the horns, is present in the cranial area. Furthermore, the dry condition of the specimen suggests that a later removal would have severely damaged the specimen, which it shows no signs of.

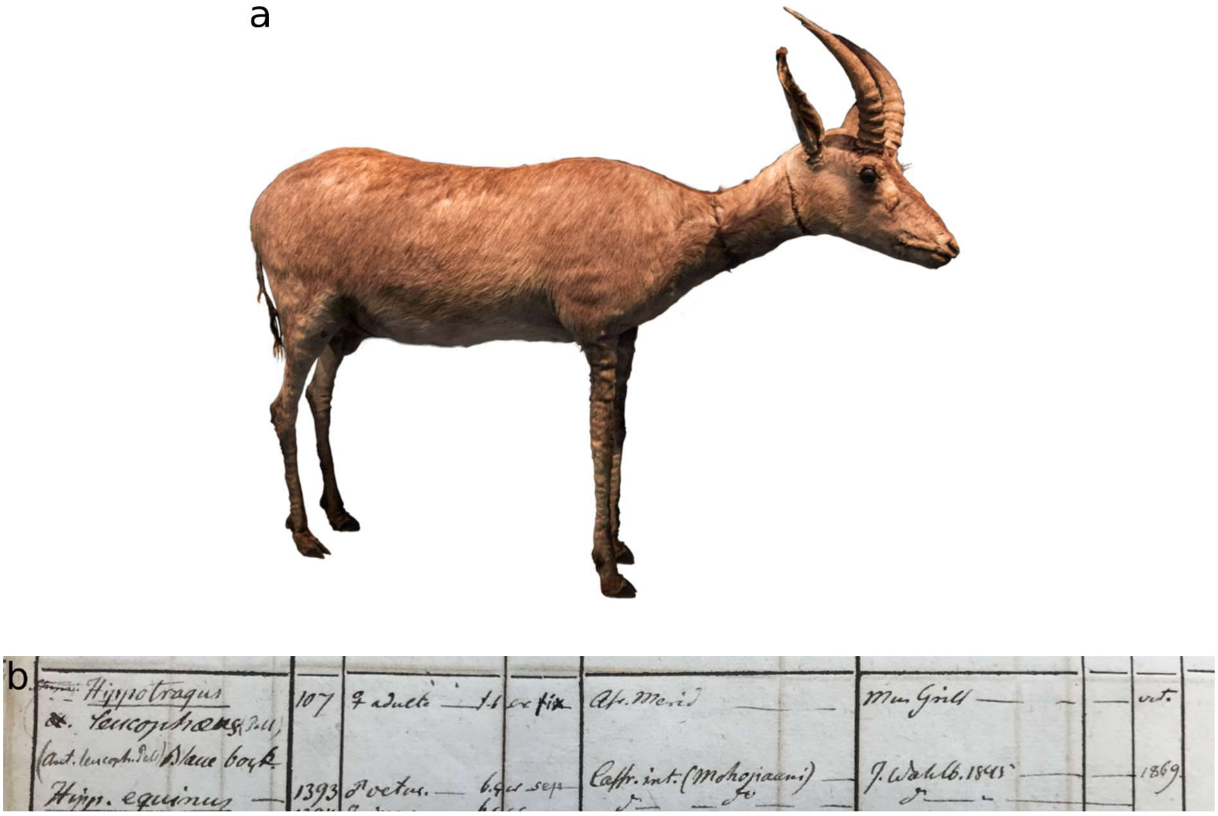

Supplementary Figure S1 (a) Subadult male blue antelope specimen of the Swedish Museum of Natural History (Naturhistoriska riksmuseet) (NRM 590107). (b) Catalogue entry of the specimen. Photo credits: Swedish Museum of Natural History.

Natural History Museum Vienna - NMW ST 715

The only known female specimen is housed in the Natural History Museum Vienna (NMW ST 715) (**Fig. 1a**). It was collected by Georg Scholl, either during his expedition to the Cape region in South Africa (1786-1788) or during subsequent years that he spent there, so that in all likelihood the specimen dates from the time between 1786 and the species‘ extinction in 1799/1800. It was first recorded in the museum in 1806. The only information on the geographical origin is a note reading ‘Caput bonae spei’ (Cape of Good Hope), but possibly this was only in reference to the Cape region in total. The Vienna specimen was first described by Kohl[3]. It has been hypothesized that the lighter neck and back might be a sign of sexual dimorphism or a difference in exposure to light[1]. Nevertheless, with only one female and three males this cannot be settled and could as well be a sex-independent intraspecific variation. No skull and hardly any osteological material are still residing inside the specimen as proven by an X-ray analysis from June 2017 (**Supplementary Fig. S2**).

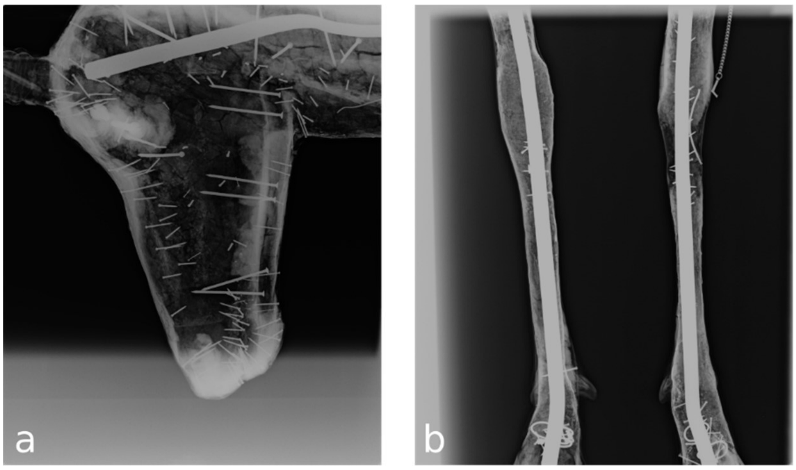

Supplementary Figure S2 (a) X-ray images of the head and (b) frontal view of the front legs of the female blue antelope specimen of the Natural History Museum Vienna (NMW ST 715) showing that no skull or skeletal material is inside the specimen (except for a phalanx on each side). See main article for a picture of the whole specimen. Photo credits: Natural History Museum Vienna.

Naturalis Biodiversity Center - RMNH.MAM.20681.a

Naturalis Biodiversity Center in Leiden (former Rijksmuseum van Natuurlijke Historie) houses skull fragments (RMNH.MAM.20681.a) (**Supplementary Fig. S3**). It has been argued by Husson & Holthuis[4] that they originate from the mounted skin (see below) of the museum (**Supplementary Fig. S5**). The specimen is referred to as a complete skull in the collection catalogue from 1887[4,5]. Perhaps the entire skull was intended to be removed from the mounted skin, but only snout fragments were taken instead. If indeed only the now available fragments had been removed, they were probably overlooked for the later catalogue of 1892[4]. The skull fragments bear the same object ID as the mounted skin, with the extension of 'b' for the mounted skin and 'a' for the skull fragments. In this study, this specimen is referred to as Leiden 1.

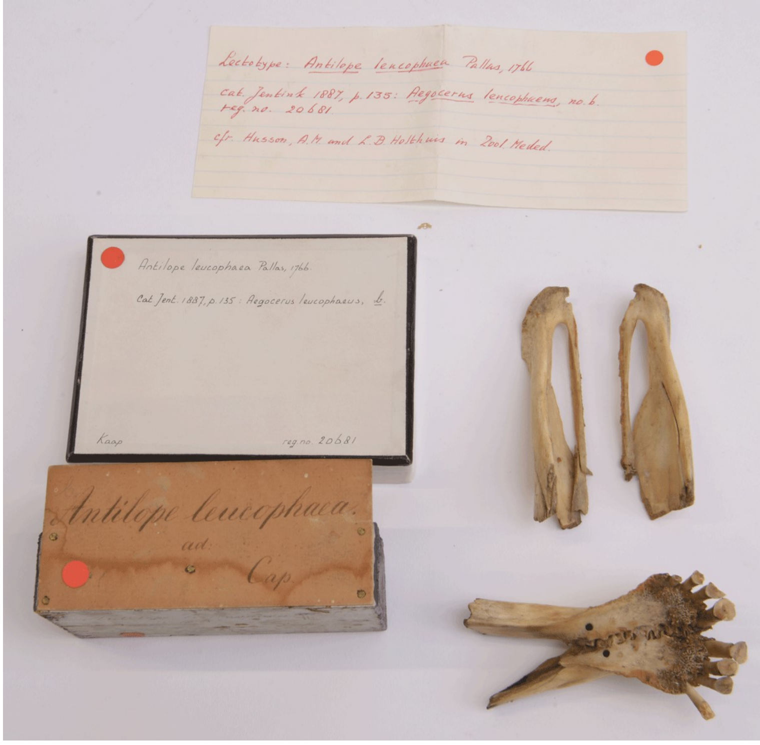

Supplementary Figure S3 Partial skull fragments of the anterior part of the mandible and premaxillary bones of a blue antelope including labels from the Naturalis Biodiversity Center in Leiden (present object ID RMNH.MAM.20681.a). It is marked as type, because it might originate from the lectotype, a mounted skin housed in the same collection (see **Supplementary Figure S5**). Photo credit: Naturalis Biodiversity Center, the Netherlands.

Museum of Evolution (University of Uppsala) - UPSZMC 78488

A pair of horns without a frontlet (UPSZMC 78488) (**Supplementary Fig. S4**) is housed in the Museum of Evolution at the University of Uppsala. These horns are most likely the remainder of a mounted skin which was dismantled sometime after 1846[1,6], possibly as late as 1886[3], 1899[7] or 1904[8].

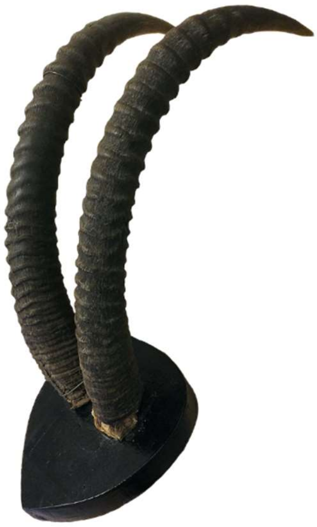

Supplementary Figure S4 Pair of horns without frontlet of a blue antelope in the Museum of Evolution at the University of Uppsala (UPSZMC 78488). Photo credit: Museum of Evolution.

Information about specimens not analyzed

Naturalis Biodiversity Center - RMNH.MAM.20681.b

The lectotype of the blue antelope is housed in the Naturalis Biodiversity Center (formerly Rijksmuseum van Natuurlijke Historie) in Leiden (RMNH.MAM.20681.b) (**Supplementary Fig. S5**). It is an adult male. In order not to confuse it with the skull fragments with the same object ID, the mounted skin is called 'a' and the skull fragments 'b'. Temminck[9] believed this to be the type of Pallas, although without giving any further explanation. This was questioned by Mohr[1]. Husson & Holthuis[4] traced the specimen's history after its arrival in Europe, and it seems very likely that it was one of the specimens (syntypes) used by Pallas to describe the species when he visited Leiden. This makes the Leiden specimen the only one with detailed information about its history and a textbook example of thorough and successful historical reconstruction based on museum archives.

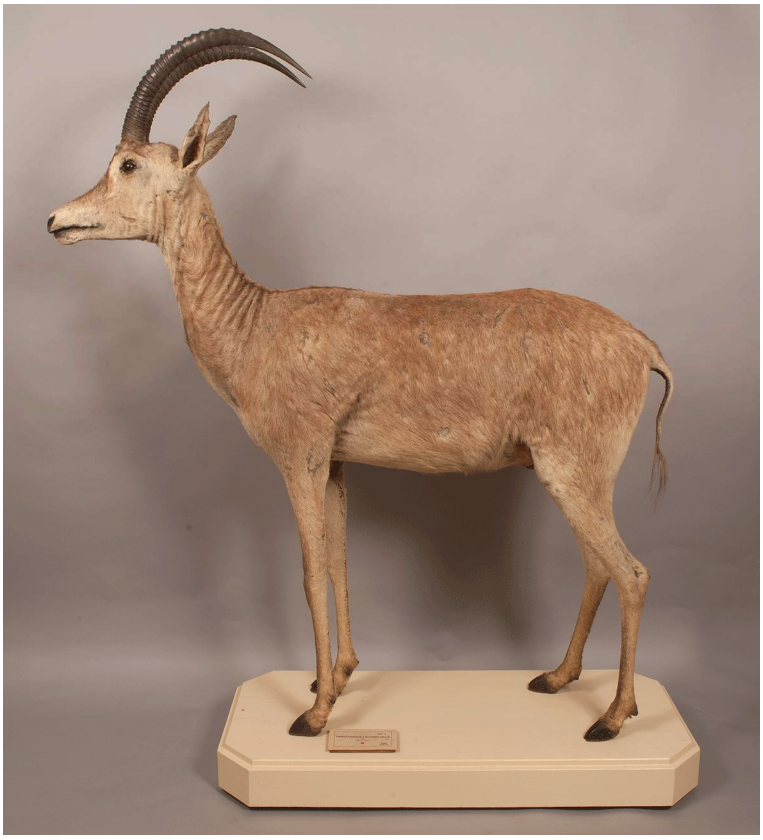

Supplementary Figure S5 A mounted skin of a male blue antelope in the Naturalis Biodiversity Center in Leiden (RMNH.MAM.20681.b). Lectotype of the species. Photo credit: Naturalis Biodiversity Center, the Netherlands.

Muséum national d'Histoire naturelle - MNHN-ZM-MO-1994-1103

The Collections d'Anatomie Comparée at the Muséum national d'Histoire naturelle in Paris owns a mounted skin of an adult male (MNHN-ZM-MO-1994-1103) (**Supplementary Fig. S6**) which was first recorded in a museum catalogue from the end of the 19th century (pers. comm. Joséphine Lésur, in charge of the Collections Ostéologiques d'Anatomie Comparée at the Muséum national d'Histoire naturelle). The entry saying it was collected by 'Delgorgue' [sic] is almost certainly incorrect, since he travelled South Africa when the blue antelope was already extinct[1,10,11]. Based on its similarity to a drawing by Robert Jacob Gordon[1], who sent blue antelope specimens to Jean Nicolas Sébastien Allamand in Leiden around 1780[10], Rookmaaker[10] considered this specimen to be the one sent to Leiden by Gordon. It might have been part of the collection of William V in The Hague from where it might have been moved to Paris in 1795[1,10,12]. Alternatively, the specimen might have been shot by François Le Vaillant in 1781[11,13]. It seems that Le Vaillant sold his collection to the Muséum national d'Histoire naturelle in Paris including one so far undescribed large gazelle ('une grande gazelle non décrite')[14]. Considering this together with the way the specimen was skinned[11], it might as well be Le Vaillant's specimen that is preserved there. It bears a resemblance to both the sketches by Gordon and Le Vaillant. The specimen is 'mounted as a mannequin'[15] and therefore does not contain any skull or other osteological material (pers. comm. Joséphine Lésur).

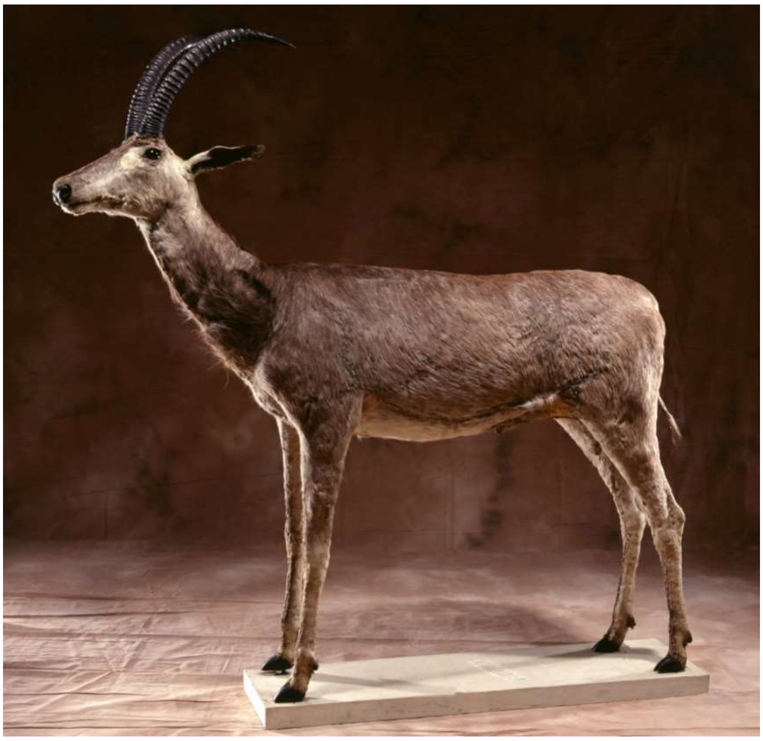

Supplementary Figure S6 Mounted skin of a male blue antelope in the Muséum national d'Histoire naturelle in Paris (MNHN-ZM-MO-1994-1103). Photo credit: MNHN - C. Lemzaouda.

Natural History Museum, London - NHMUK GERM 636e

The Natural History Museum in London houses a frontlet with horns (NHMUK GERM 636e) that is labeled as belonging to the blue antelope (**Supplementary Fig. S7**). It is mentioned by Lydekker[16] as 'provisionally referred to this species' and it was mentioned by Sclater & Thomas[7] as having 'been long in the Museum'. Based on similarities in horn length and the number of rings, it is possible that this specimen might be the remainder of a skin bought by Thomas Pennant in Amsterdam[1,8]. Alternatively, it might have been bought for the British Museum by William Elford Leach at an auction in 1819[6].

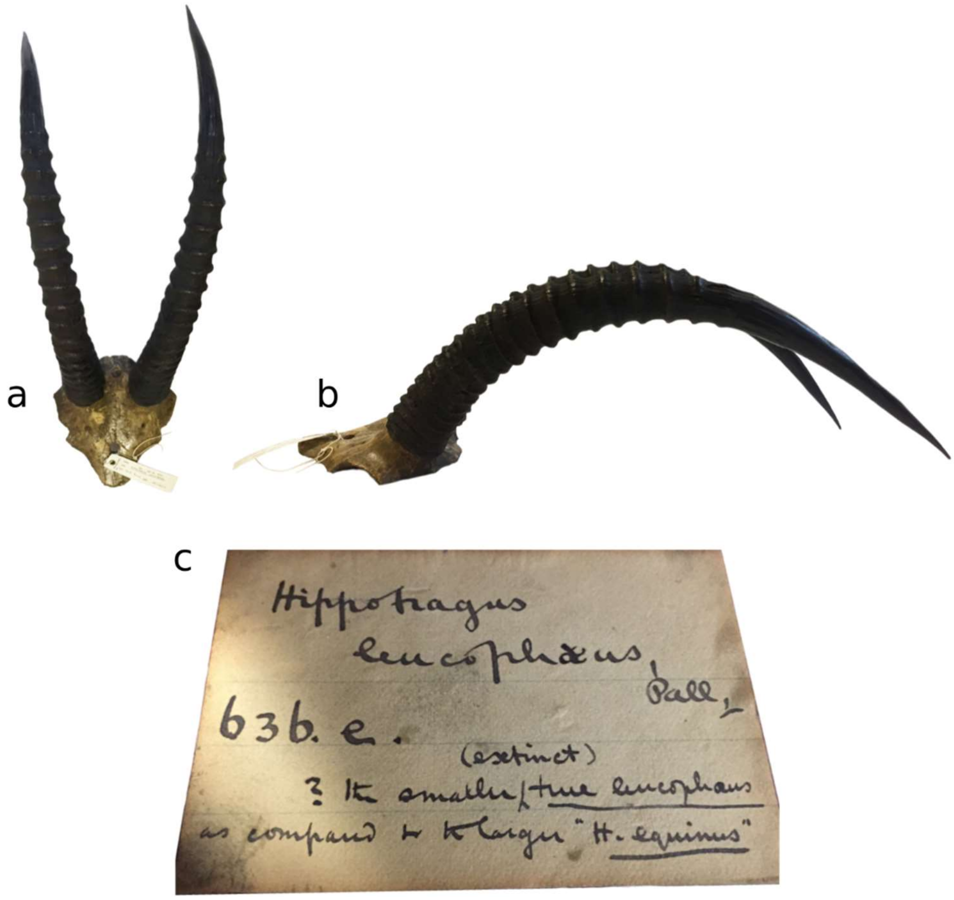

Supplementary Figure S7 (a & b) Frontlet with horns from the Natural History Museum London (NHMUK GERM 636e). (c) Entry card of the specimen labeling it as blue antelope. Photo credits: Courtesy of the Trustees of the Natural History Museum, London.

Royal Belgian Institute of Natural Sciences - RBINS 3785

The Royal Belgian Institute of Natural Sciences in Brussels owns either a skull (collection catalogue) or a pair of horns (collection entry) (RBINS 3785). Due to collection renovation the nature of the specimen could not be determined. It was donated to the museum by Dr Louis Giltay (1903-1937) together with six pairs of horns of ungulates (pers. comm. Olivier S.G. Pauwels, curator of the Recent Vertebrates Collection at the Royal Belgian Institute of Natural Sciences). In the catalogue, it is tentatively identified as 'cf. *leucophaeus*'. Since Dr Gilay lived long after the blue antelope became extinct, he cannot be the original collector, and it remains unknown how the specimen came into his possession.

Museum für Naturkunde, Berlin - ZMB MAM 8855 & ZMB MAM 8860

The specimens ZMB MAM 8855 (**Supplementary Fig. S8**) and ZMB MAM 8860 (**Supplementary Fig. S9**) in the Museum für Naturkunde in Berlin were formerly labeled as blue antelopes. The cranium ZMB MAM 8855 of a female from South Africa is currently labeled as a sable, whereas the cranium with mandible of a male from Sudan ZMB MAM 8860 is labeled as roan, which is likely if its location (Sudan) is correct. Furthermore, its documented collector Georg August Schweinfurth was born after the extinction of the blue antelope and is so far not known to have travelled in South Africa. It was originally part of the Anatomical Collection of the Friedrich-Wilhelms-Universität zu Berlin (now Humboldt University of Berlin) (pers. comm. Steffen Bock, collection manager at the Mammal Collection of the Museum für Naturkunde, Berlin, 2016-2018). It is unknown how long these two specimens have been part of the Berlin collections (pers. comm. Steffen Bock), although ZMB MAM 8860 might have been there since 1897.

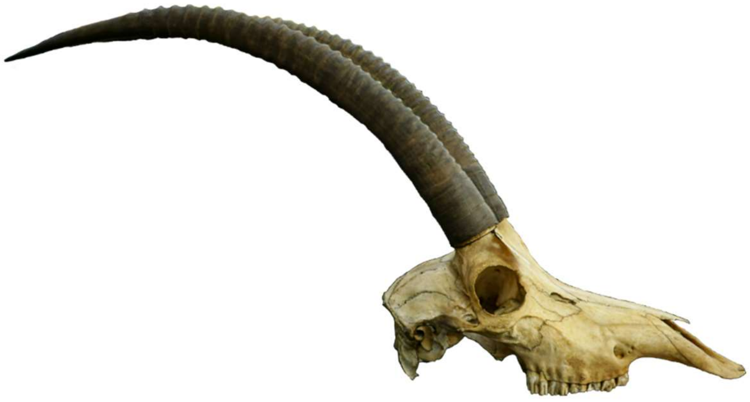

Supplementary Figure S8 The cranium of a female specimen once labeled as blue antelope (ZMB MAM 8855) from the Mammal Collection of the Museum für Naturkunde, Berlin. The specimen is currently labeled as sable. Photo credit: E. Hempel, courtesy: Mammal Collection, Museum für Naturkunde, Berlin.

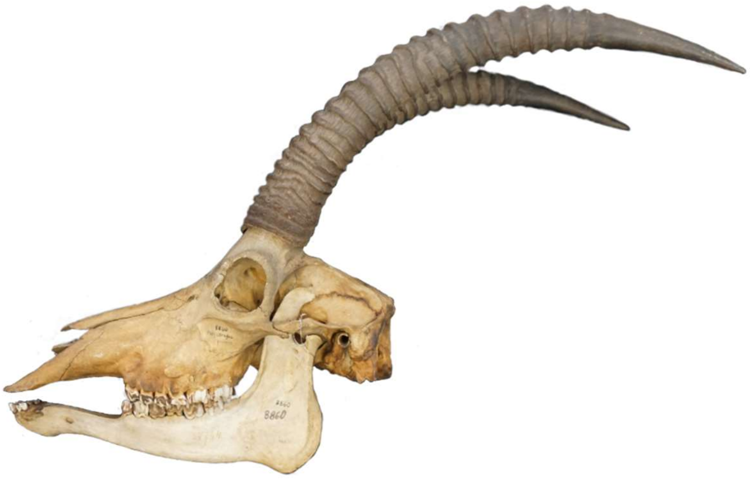

Supplementary Figure S9 The cranium and mandible of a male specimen formerly labeled as blue antelope (ZMB MAM 8860) from the Mammal Collection of the Museum für Naturkunde, Berlin. The specimen is currently labeled as roan. Photo credit: E. Hempel, courtesy: Mammal Collection, Museum für Naturkunde, Berlin.

Available information about analyzed specimens - Roan antelope (*Hippotragus equinus*)

Bell Pettigrew Museum - BPM 2234

The Bell Pettigrew Museum at the University of St Andrews owns a frontlet with horns (BPM 2234) (**Supplementary Fig. S10**) with no further information attached to it.

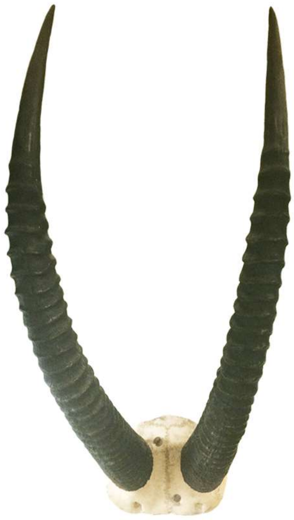

Supplementary Figure S10 The frontlet with horns of a roan at the Bell Pettigrew Museum at the University of St Andrews (BPM 2234). Photo credit: University of St Andrews.

Muséum national d'Histoire naturelle - MNHN-ZM-AC-1896-100

A cranium from the Muséum national d'Histoire naturelle in Paris (MNHN-ZM-AC-1896-100) (**Supplementary Fig. S11**) labeled as blue antelope from Senegal was donated to the museum by Houdelot(?). It is known from a catalogue from the end of the 19th century (pers. comm. Joséphine Lésur).

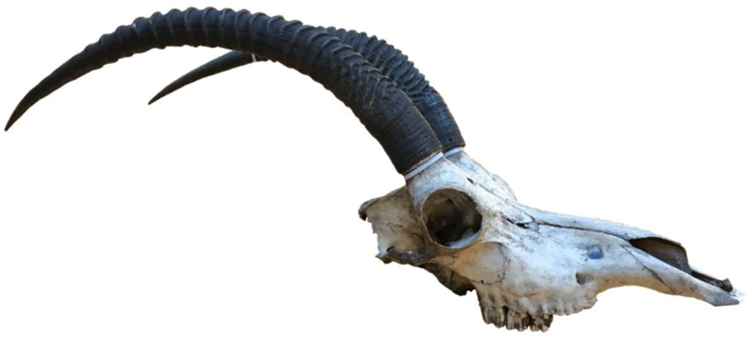

Supplementary Figure S11 The cranium of a roan from the Muséum national d'Histoire naturelle in Paris (MNHN-ZM-AC-1896-100). Photo credit: Muséum national d'Histoire naturelle, Collections d'Anatomie Comparée.

Museum für Naturkunde, Berlin - ZMB MAM 8859

The cranium ZMB MAM 8859 (**Supplementary Fig. S12**) at the Museum für Naturkunde in Berlin was formerly identified as a blue antelope, but then relabeled as a roan, possibly after a revision of Mohr[1]. It is without locality information; sex and entry date in the collection are unknown.

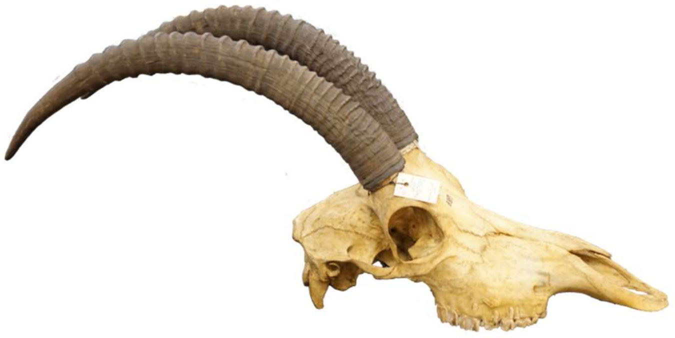

Supplementary Figure S12 The cranium of a roan (ZMB MAM 8859) from the Mammal Collection of the Museum für Naturkunde, Berlin. Photo credit: E. Hempel, courtesy: Mammal Collection, Museum für Naturkunde, Berlin.

Available information about analyzed specimens - Sable antelope (*Hippotragus niger*)

The Hunterian (University of Glasgow) - GLAHM:Z4884

A cranium with mandible but no horn sheaths (**Supplementary Fig. S13**) exists in the Hunterian at the University of Glasgow, where it was first suspected to be a blue antelope specimen by Broom[17] in 1949. Not much is known about its provenance, and it is not mentioned in any catalogue[1]. According to a former employee it had already been in the old zoological museum at the University of Glasgow and was therefore obtained before 1923, and probably before 1902[1]. Broom[17], Mohr[1] and Groves & Westwood[18] assigned the Glasgow skull to the blue antelope after taking various measurements, whereas Klein[19] considered it more likely to be sable.

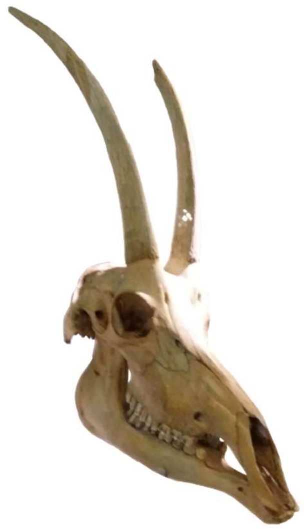

Supplementary Figure S13 The cranium with mandible of a sable (GLAHM:Z4884) without horn sheaths at the Hunterian at the University of Glasgow. Photo credit: E. Hempel, courtesy: the Hunterian, University of Glasgow.

Iziko Museums of South Africa, Terrestrial Vertebrate Collection -

SAM ZM 40759

The Terrestrial Vertebrate Collection at the Iziko Museums of South Africa in Cape Town owns a frontlet with a pair of horns (SAM ZM 40759) (**Supplementary Fig. S14**) that was formerly owned privately and was donated to the collection in 1989 (pers. comm. Denise Hamerton, curator of the Terrestrial Vertebrate Collection at the Iziko Museums of South Africa). At least according to the literature, it seems that it has never been examined thoroughly. Ozinsky[20] only mentions that it was identified by Dr Richard Klein, but does not go into detail. Some measurements are given by Rookmaaker[6].

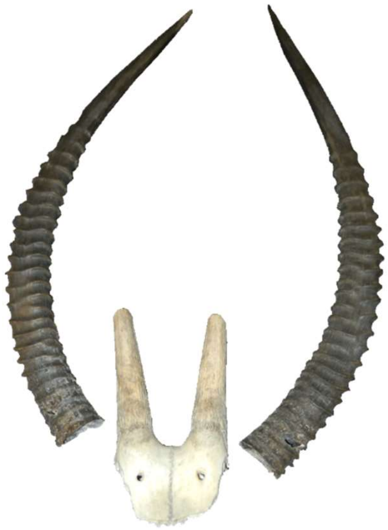

Supplementary Figure S14 Frontlet with horns of a sable (SAM ZM 40759) at the Terrestrial Vertebrate Collection at the Iziko Museums of South Africa in Cape Town. Photo credit: J. T. Faith, courtesy: Terrestrial Vertebrate Collection, Iziko Museums of South Africa.

Naturalis Biodiversity Center - ZMA.MAM.18623

The cranium with mandible in the Naturalis Biodiversity Center in Leiden (ZMA.MAM.18623) (**Supplementary Fig. S15**) was transferred to the collection from the Zoölogisch Museum in Amsterdam. The skull was tentatively identified as 'cf. *leucophaeus*' by Erdbrink[21] and also considered as such by Rookmaaker[6] and, if it is a female, also by Groves & Westwood[18]. There is no further provenance information. A skin with horns of a blue antelope was donated by Jacob Abraham Uytenhage De Mist to the Hollandsche Maatschappij der Wetenschappen in Haarlem in 1805 (see Lost specimens). It was presumed that the skull ZMA.MAM.18623 might be the remains of this specimen, but there are no further records about it[6]. In this study, this specimen is referred to as Leiden 2.

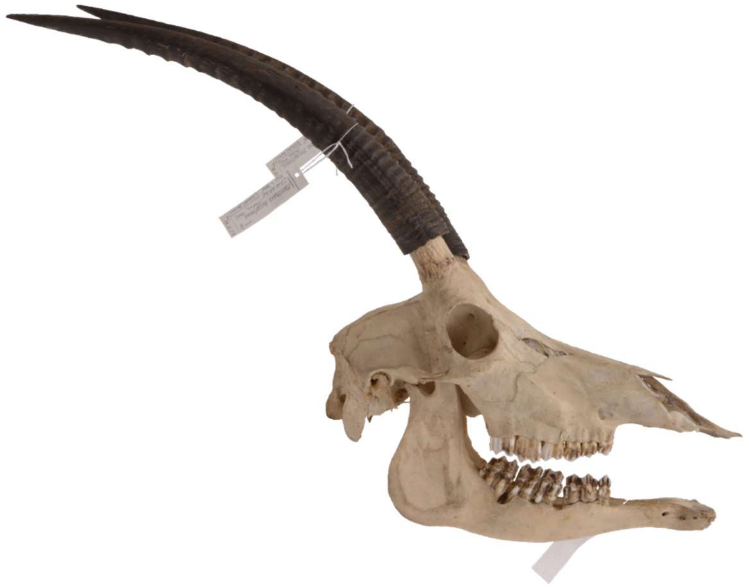

Supplementary Figure S15 The cranium with mandible of a sable (ZMA.MAM.18623) at the Naturalis Biodiversity Center in Leiden. Photo credit: Naturalis Biodiversity Center, the Netherlands.

Lost specimens

In **Supplementary Table S1** all specimens are listed that might have been blue antelope specimens, but which are believed to be lost today.

Supplementary Table S1 List of potential blue antelope specimens that are considered lost today. See Mohr[1], Rookmaaker[6] and Renshaw[8] for more detailed information.

ID	Museum	Specimen type
149	Albany Museum (Grahamstown)/Amathole Museum (King William's Town)	pair of horns
1356	Royal College of Surgeons (London)	cranium with horns & horn sheaths, no mandible, no premaxillary bones
?	Haarlem	skin with horns
?	Skin collected by Thomas Pennant (probably same as the London frontlet with horns)	skin with horns
?	Museum für Naturkunde (Berlin) (?)	skin
?	Natural History Museum (London)	frontlets with horns

Albany Museum, Republic of South Africa, pair of horns

Lydekker[22] refers to a 'Report of the Albany Museum for the year 1901' in which a pair of horns of the blue antelope is mentioned. Mohr[1] quotes a letter by Prof Eloff from 1966 stating that a fire destroyed a complete wing of the museum and that it is likely that the horns were destroyed as well. Rookmaaker[6] reports that W. H. Holleman, deputy director of the museum, confirmed that no *H. leucophaeus* horns are housed in the museum. Today, most of the Albany Museum collection has been relocated to the Amathole Museum in King William's Town (pers. comm. Paulus Janse, exhibitions officer at the Albany Museum, Grahamstown). The Amathole Museum provided us with a list of all current *Hippotragus* specimens. In this list, nine specimens are identified only on the genus level. It could be worth investigating if one of them might be the missing blue antelope specimen.

Royal College of Surgeons, London, United Kingdom, cranium

Mohr[1] found that the cranium without mandible was destroyed during a bombing in 1941. According to J. Dolson (in Mohr[1]) it was listed as '1356 in the catalogue of Osteology of Vertebrated Animals, 1884. It is stated to have been in the museum before 1861 but the

donator is unknown. The specimen consisted of the horns only.' A picture in Mohr[1], however, shows a cranium with horn sheaths but lacking the mandible and premaxillary bones. Renshaw[8] considered it to be a blue antelope whereas Mohr[1] identified it as roan.

The specimen does not exist in the collection today, but a lot of osteological material has been transferred to the Natural History Museum in London (pers. comm. Carina Phillips, head of museum collections at the Royal College of Surgeons, London). It is not on the list of transferred items held by the Royal College of Surgeons, but this record is known to be incomplete (pers. comm. Carina Phillips). Although the specimen was likely lost in the bombing during World War II, it needs to be determined if it still exists in the Natural History Museum in London. The Natural History Museum does not have a specimen by that ID in its collection, but suffered losses in a mammal gallery during World War II as well, resulting in specimens without labels which are now only labelled as *Hippotragus* sp. (pers. comm. Roberto Portela Miguez, senior curator in charge of mammals at the Natural History Museum, London).

Hollandsche Maatschappij der Wetenschappen in Haarlem, skin with horns

Rookmaaker[6] writes about a skin with horns that was given to Martinus van Marum at the Hollandsche Maatschappij der Wetenschappen in Haarlem, Netherlands, by Jacob Abraham Uytenhage de Mist in 1805. See Rookmaaker[6] for further details on the potential fate of this specimen.

Skin collected by Thomas Pennant, skin with horns

According to Renshaw[8] Thomas Pennant acquired a skin with horns still attached in Amsterdam before 1781. Due to similarities in the number of horn rings and length, Renshaw[8] considers it possible that the pair of horns at the Natural History Museum in London might be the remainder of that skin. Mohr[1] acknowledges the similarities, but points out that these cannot be seen as conclusive evidence that it is the same individual.

Museum für Naturkunde, Berlin(?), skin

Lichtenstein[23] mentions a skin that he used for his description, but fails to mention if he possessed this skin or saw it elsewhere, although Renshaw[8] stated 'In 1799 a *leucophaeus* was shot and sent to Berlin.' Martin Heinrich (or Hinrich) Carl Lichtenstein was present at an auction in London 1819, where a blue antelope was sold[6]. If that specimen really was a blue antelope, the rather low price and therefore disinterest of Lichtenstein[6], who was at the time director of the Zoological Museum in Berlin (a predecessor of today's Museum für Naturkunde), could be explained with Berlin already owning a blue antelope skin. Unfortunately, the Museum für Naturkunde, Berlin presently has no records of any skins of the blue antelope.

Natural History Museum, London, frontlets with horns

Lydekker[22] stated that the 'British Museum also possesses a couple of frontlets with horns believed to belong to the blaauwbok.' Whether he exaggerated and actually meant the one known frontlet with horns or if indeed there have been (or possibly still are) more in the collection remains to be determined.

Supplementary Table S2 Sequencing yield from eleven next generation sequencing libraries of the received potential blue antelope samples (*Hippotragus leucophaeus*) after mapping to the complete mitochondrial genomes of *H. leucophaeus* (this study), *H. niger* (NC_020713[24]) and *H. equinus* (NC_020712[24]).

**Sample ID**	**Run #**	**Total reads**	**<30bp**	**reads for mapping**	***H. leucophaeus* mapped reads**	***H. leucophaeus* unique reads**	***H. leucophaeus* mapped bp**	***H. niger* mapped reads**	***H. niger* unique reads**	***H. niger* mapped bp**	***H. equinus* mapped reads**	***H. equinus* unique reads**	***H. equinus* mapped bp**
**NRM 590107**	test #1	2,452,025	2,046,097										
	after Pippin Prep # 1	13,749,760	4,680,667										
	**total**	**16,201,785**	**6,726,764**	**9,475,021**	**44,749**	**40,379**	**1,720,740**	**15,413**	**14,025**	**585,999**	**11,372**	**10,29**	**427,823**
**NMW ST 715**	test #1	3,511,024	3,460,636										
	after Pippin Prep # 1	219,104	103,915										
	capture #1	2,065,658	911,358										
	after Pippin Prep # 2	443,192	213,389										
	capture #2	7,126,935	6,208,164										
	after Pippin Prep # 3	2,062,881	985,384										
	after Pippin Prep & re-amplification #1	4,539,744	1,916,544										
	after Pippin Prep & re-amplification #2	6,138,573	2,608,519										
	**total**	**26,107,111**	**16,407,909**	**9,699,202**	**1,160,000**	**2,601**	**102,473**	**437,385**	**1,015**	**40,14**	**341,173**	**797**	**31,268**

**UPSZMC 78488 (skin)**	test #1	2,558,408	2,160,289										
	capture #1	508,031	30,352										
**UPSZMC 78488 (bone)**	test #1	2,462,689	772,895										
	capture #1	2,397,449	45,069										
	**total**	**7,926,577**	**3,008,605**	**4,917,972**	**2,563,686**	**7,936**	**292,509**	**1,769,784**	**2,81**	**103,922**	**1,114,115**	**2,142**	**78,653**
**RMNH.MAM.20681.a**	test #1	2,398,711	595,675										
	capture #1	6,289,940	197,18										
	test #2	7,011,417	1,559,919										
	**total**	**15,700,068**	**2,352,774**	**13,347,294**	**5,422,744**	**1,078**	**64,347**	**1,980,112**	**380**	**21,796**	**1,152,431**	**234**	**13,281**
**GLAHM:Z4884**	test #1	2,657,956	516,753										
	capture #1	5,132,954	78,804										
	**total**	**7,790,910**	**595,557**	**7,195,353**	**1,928,373**	**372**	**20,979**	**4,760,459**	**1,122**	**67,317**	**1,675,095**	**259**	**14,259**
**SAM ZM 040759**	test #1	2,323,185	820,716										
	capture #1	4,646,459	141,455										
	**total**	**6,969,644**	**962,171**	**6,007,473**	**1,851,358**	**252**	**14,45**	**4,287,014**	**623**	**36,784**	**1,422,835**	**192**	**10,882**
**ZMA.MAM.18623**	test #1	3,226,821	693,971										
	capture #1	15,149,640	5,099,924										
	**total**	**18,376,461**	**5,793,895**	**12,582,566**	**1,522,773**	**319**	**17,543**	**3,374,044**	**498**	**28,29**	**1,336,479**	**261**	**14,082**
**ZMB MAM 8859**	test #1	2,739,865	1,052,628										
	capture #1	3,296,732	88,953										
	**total**	**6,036,597**	**1,141,581**	**4,895,016**	**1,413,743**	**430**	**19,395**	**1,211,164**	**400**	**18,212**	**3,141,262**	**1,698**	**83,455**

**MNHN-ZM-AC-1896-100**	test #1	2,341,377	397,381										
	capture #1	2,410,438	18,017										
	**total**	**4,751,815**	**415,398**	**4,336,417**	**855,063**	**568**	**31,55**	**808,538**	**529**	**28,875**	**2,089,398**	**2,039**	**119,365**
**BPM 2234**	test #1	2,713,298	317,55										
	capture #1	16,367,563	154,186										
	**total**	**19,080,861**	**471,736**	**4,327,763**	**4,327,763**	**870**	**54,621**	**3,496,901**	**789**	**49,653**	**15,140,570**	**3,409**	**225,44**

Supplementary Table S3 Pooling strategy for hybridization capture 1. Samples were pooled in equimolar amounts according to their estimated mitochondrial endogenous content. Samples with an estimated mitochondrial endogenous content of 0 were set to 0.0001 (marked with *). All samples from this project were mapped to the *H. leucophaeus* (MF043256[24,25]), *H. niger* (NC_020713[23,24]) and *H. equinus* (NC_020712[23,24]) mitochondrial genomes. Only the estimated endogenous content for the reference with the highest mapping success is shown. For pooling, the highest resulting endogenous content was used. Samples 1-18, exBlks1-5 and liBlk1-5 are from different projects (exBlk = extraction blank, liBlk = library blank). [#] The 'Amount of DNA added' and the desired 'Estimated amount of target DNA added to pool' was halved for the Uppsala sample (UPSZMC 78488) since two libraries from the same sample were pooled. [§] Percentage of filtered reads mapped to reference with highest mapping success. [$] In case of two TapeStation peaks the mean was used as average fragment size.

Sample	Concen-tration [ng/µl]	Average fragment size [bp]	Estimated molarity [nM]	Estimated target content[§]	Amount of DNA added [ng]	Estimated amount of target DNA added to pool [ng]
NMW ST 715	3.32	155	32.45	0.0014 %	6.03	0.0086
RMNH.MAM.20681.a	2.94	181	24.61	0.0030 %	3.39	0.0100
UPSZMC 78488 bone	1.3	174	11.32	0.0022 %	2.20[#]	0.0048[#]
UPSZMC 78488 skin	0.0285	157	0.28	0.2967 %	0.01[#]	0.0044[#]
GLAHM:Z4884	0.452	182	3.76	0.0199 %	0.51	0.0101
SAM ZM 40759	1.23	175	10.65	0.0040 %	2.42	0.0097
ZMA.MAM.18623	4.28	179	36.23	0.0006 %	16.85	0.0099
BPM 2234	2.18	209	15.80	0.0063 %	1.84	0.0116
MNHN-ZM-AC-1896-100	0.278	197	2.14	0.0760 %	0.14	0.0109
ZMB MAM 8859	0.254	168	2.29	0.0516 %	0.18	0.0093
Sample 1	6.96	175	60.26	0.0003 %	37.31	0.0097
Sample 2	9.22	178	78.48	0.0001 %*	98.68	0.0099
Sample 3	16.1	172	141.83	0.0001 %*	95.36	0.0095
Sample 4	9.74	177	83.38	0.0004 %	22.60	0.0098
Sample 5	17.6	166	160.64	0.0001 %	66.52	0.0092
Sample 6	12.7	166[$]	115.92	0.0001 %*	92.03	0.0092
Sample 7	6.46	176	55.61	0.0014 %	6.76	0.0098
Sample 8	10.1	167	91.63	0.0001 %*	92.58	0.0093
Sample 9	12.9	173	112.98	0.0001 %*	95.91	0.0096
Sample 10	51	140	551.95	0.0001 %*	77.62	0.0078
Sample 11	15.6	163	145.01	0.0001 %*	90.37	0.0090

Sample 12	22	167	199.60	0.0001 %*	92.58	0.0093
Sample 13	18.1	169	162.27	0.0001 %*	93.69	0.0094
Sample 14	17.3	166	157.90	0.0001 %*	92.03	0.0092
Sample 15	11.4	164	105.32	0.0001 %*	90.92	0.0091
Sample 16	93	140	1006.49	0.0001 %*	77.62	0.0078
Sample 17	11.5	170	102.50	0.0001 %	68.46	0.0094
Sample 18	0.11	175	0.95	0.0736 %	0.13	0.0097
ExBlk08082018	21.8	155	213.10	0.0001 %*	17.19	0.0017
ExBlk28082018	7.26	161	68.32	0.0001 %*	17.85	0.0018
ExBlk24102018	4.52	156	43.90	0.0007 %	2.53	0.0017
ExBlk1	1.75	159	16.68	0.0011 %	1.59	0.0018
ExBlk 2	13.2	155	129.03	0.0001 %*	17.19	0.0017
ExBlk 3	13.9	156	135.00	0.0001 %*	17.30	0.0017
ExBlk 4	31.6	152	314.99	0.0001 %*	16.85	0.0017
ExBlk 5	34.4	156	334.11	0.0001 %*	17.30	0.0017
LiBlk15082018	17	145	177.64	0.0001 %*	16.08	0.0016
LiBlk05092018	24.4	140	264.07	0.0001 %*	15.52	0.0016
LiBlk07112018	13.5	148	138.21	0.0001 %*	16.41	0.0016
LiBlk 1	13.6	151	136.46	0.0001 %*	16.74	0.0017
LiBlk 2	10.2	139	111.18	0.0001 %*	15.41	0.0015
LiBlk 3	11.3	151	113.39	0.0001 %*	16.74	0.0017
LiBlk 4	14.2	163	131.99	0.0001 %*	18.07	0.0018
LiBlk 5	12.4	152	123.60	0.0001 %*	16.85	0.0017

Supplementary Table S4 Pooling strategy for hybridization capture 2 – NMW ST 715 after Pippin Prep treatment. The sample NMW ST 715 was pooled again after being treated on the Pippin Prep on a run of a different project, which was processed in the same way. Samples were pooled in equimolar amounts according to their estimated mitochondrial endogenous content. Samples with an estimated mitochondrial endogenous content of 0 were set to 0.0001 (marked with *). NMW ST 715 was mapped to the *H. leucophaeus* (MF04325624[25]), *H. niger* (NC_02071323[24]) and *H. equinus* (NC_02071223[24]) mitochondrial genomes. Only the estimated endogenous content for the reference with the highest mapping success is shown. For pooling, the highest resulting endogenous content was used. Samples 6-10, exBlks2-4 and liBlk2-4 are from a different project (exBlk = extraction blank, liBlk = library blank). [§] Percentage of filtered reads mapped to reference with highest mapping success. [+] The concentration of NMW ST 715 was too low to be measured after the Pippin Prep treatment and was therefore estimated to be the same as for the other sample processed on the Pippin Prep (NRM 590107) to estimate the molarity. [&] For the endogenous content the same percentage was assumed as for the untreated sample. [$] In case of two TapeStation peaks the mean was used as average fragment size.

**Sample**	**Concen-tration [ng/µl]**	**Average fragment size [bp]**	**Estimated molarity [nM]**	**Estimated target content[§]**	**Amount of DNA added [ng]**	**Estimated amount of target DNA added to pool [ng]**
NMW ST 715 after Pippin Prep	0.382[+]	213	2.72	0.0010 %[&]	5.90	0.0059
Sample 6	12.7	166[$]	115.92	0.0001 %*	46.02	0.0046
Sample 7	6.46	176	55.61	0.0014 %	3.38	0.0049
Sample 8	10.1	167	91.63	0.0001 %*	46.29	0.0046
Sample 9	12.9	173	112.98	0.0001 %*	47.96	0.0048
Sample 10	51	140	551.95	0.0001 %*	38.81	0.0039
ExBlk 2	13.2	155	129.03	0.0001 %*	17.19	0.0017
ExBlk 3	13.9	156	135.00	0.0001 %*	17.30	0.0017
ExBlk 4	31.6	152	314.99	0.0001 %*	16.85	0.0017
LiBlk 2	10.2	139	111.18	0.0001 %*	15.41	0.0015
LiBlk 4	14.2	163	131.99	0.0001 %*	18.07	0.0018
LiBlk 5	12.4	152	123.60	0.0001 %*	16.85	0.0017

Supplementary Table S5. Sequences from GenBank used for the mitochondrial diversity comparison using pairwise distances.

Species	Sequences used in comparison	References
Alces alces	KP405229, MF784597, MF784598, MF784599, MF784600, MF784601, MF784602, MF784603, MF784604, MK644889, MK644890, MK644891, MK644892, MK644893, MK644894, MK644895, MK644896, MK644897, MK644898, MK644899, MK644900, MK644901, MK644902, MK644903, MK644904, MK644905, MK644906, MK644907, MK644908, MK644909, MK644910, MK644911, MK644912, MK644913, MK644914, MK644915, MK644916, MK644917, MK644918, MK644919, MK644920, MK644921, MK644922, MK644923 (4x), MK644924 (2x), MK644925 (5x), MK644926 (2x), MK644927 (11x), MK644928 (2x), NC_020677	[24,26–28]
Bison bison	GU946976, GU946977, GU946978, GU946979, GU946980, GU946981, GU946982, GU946983, GU946984, GU946985, GU946986, GU946987, GU946988, GU946989, GU946990, GU946991, GU946992, GU946993, GU946994, GU946995, GU946996, GU946997, GU946998, GU946999, GU947000, GU947001, GU947002, GU947003, GU947004, GU947005, GU947006, JN632601, NC_012346	[24,29,30]
Bison bonasus	JN632602, KY055664 (12x)	[24,31]
Equus przewalskii	HQ439484, JN398402, JN398403, KT368742, KT368743, KT368744, KT368745, KT368746, KT368747, KT368748, KT368749, KT368750, KT368751, KT368752, KT368753, KT368754, KT368755, KT368756	[32–34]
Oryx dammah	JN632677, MT248292, MT248293, MT248294, MT248295, MT248296, MT248297	[24,35]
Syncerus caffer	JQ235505, JQ235506, JQ235507, JQ235508, JQ235509, JQ235510, JQ235511, JQ235512, JQ235513, JQ235514, JQ235515, JQ235516, JQ235517, JQ235518, JQ235519, JQ235520, JQ235521, JQ235522, JQ235523, JQ235524, JQ235525, JQ235526, JQ235527, JQ235528, JQ235529, JQ235530, JQ235531, JQ235532, JQ235533, JQ235534, JQ235535, JQ235536, JQ235537,JQ235538, JQ235539, JQ235540, JQ235541, JQ235542, JQ235543, JQ235544, JQ235545, JQ235546, JQ235547, NC_020617	[36]

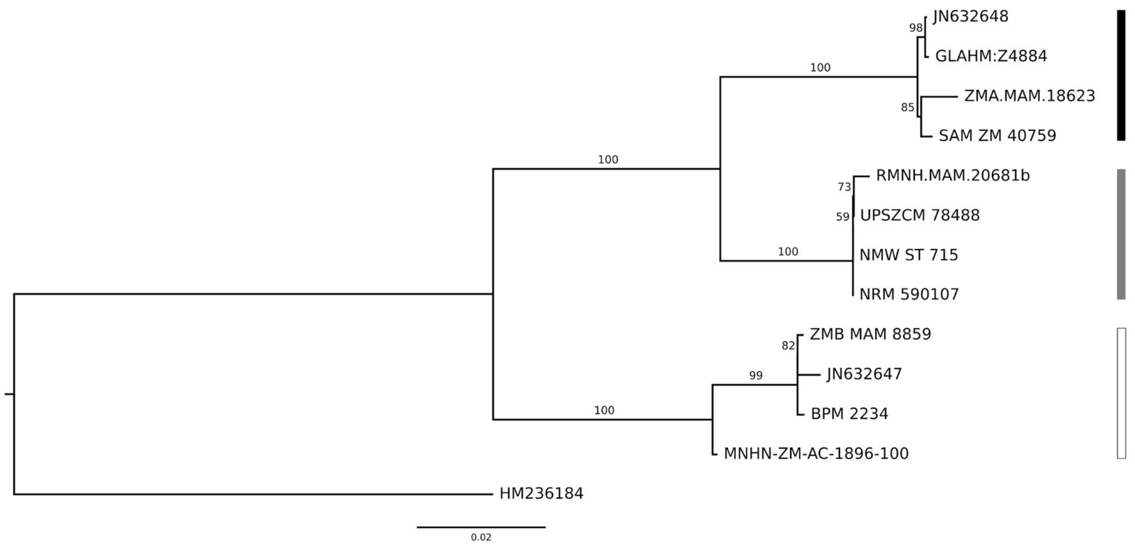

Supplementary Figure S16 Maximum-likelihood tree based on the mitochondrial genome alignment excluding the control region (15,493 bp in length) using the mitochondrial genome of *Hippotragus niger* (NC_020713[24]) as reference (black: sable, white: roan, grey: blue antelope), generated using RaxML 8.2.10 with GTR+G[37]. Bootstrap support of 100 replicates is shown on the branches. *Ovis aries musimon* (HM236184[38]) was used as outgroup.

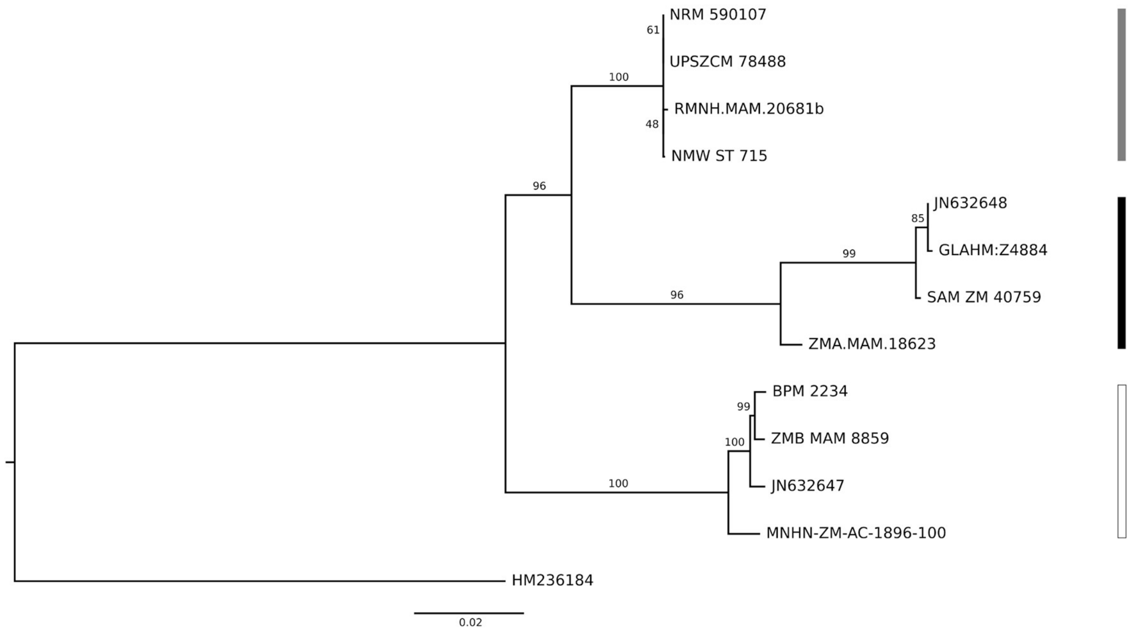

Supplementary Figure S17 Maximum-likelihood tree based on the mitochondrial genome alignment excluding the control region (15,477 bp in length) using the mitochondrial genome of *Hippotragus equinus* (NC_020712[24]) as reference (black: sable, white: roan, grey: blue antelope), generated using RaxML 8.2.10 with GTR+G[37]. Bootstrap support of 100 replicates is shown on the branches. *Ovis aries musimon* (HM236184[38]) was used as outgroup.

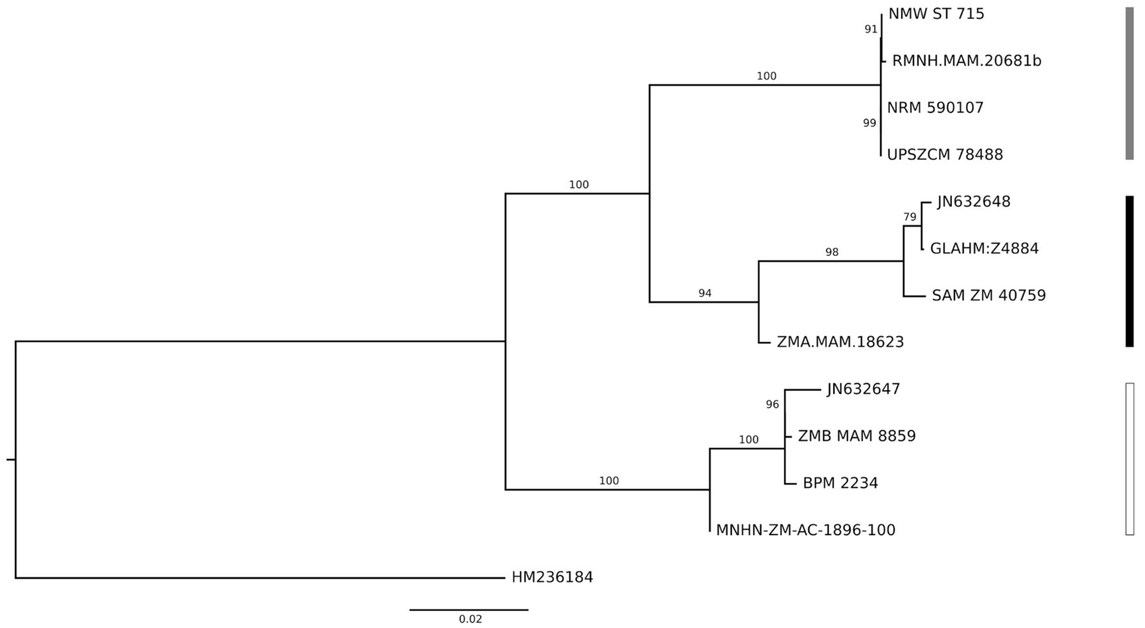

Supplementary Figure S18 Maximum-likelihood tree based on the mitochondrial genome alignment excluding the control region (15,471 bp in length) using the mitochondrial genome of *Hippotragus leucophaeus* (this study) as reference (black: sable, white: roan, grey: blue antelope), generated using RaxML 8.2.10 with GTR+G[37]. Bootstrap support of 100 replicates is shown on the branches. *Ovis aries musimon* (HM236184[38]) was used as outgroup.

Supplementary Table S6 Number of iterations, resulting contigs, resulting number of reads before and after duplicate removal with samtools rmdup v0.1.19[39] and uncorrected length of consensus sequences for each MITObim[40,41] run with different mismatch values. Bait sequences: *Hippotragus niger* (NC_020713[24]), *Hippotragus equinus* (NC_020712[24]) and *Capra hircus* (KR059146[42]).

**bait sequence**	**mismatch value**	**iterations**	**read pool**	**contigs**	**total number of reads before duplicate removal**	**total number of reads after duplicate removal**	**length - consensus sequence uncorrected for circularity**
Hippotragus niger mitochondrial genome	0	26	24287	1	24414	14192	16561
Hippotragus niger mitochondrial genome	1	22	32840	1	34034	18006	16588
Hippotragus niger mitochondrial genome	2	22	33279	1	34658	18184	16588
Hippotragus niger mitochondrial genome	3	21	33349	1	34638	18131	16588
Hippotragus niger mitochondrial genome	4	21	33382	1	34677	18135	16588
Hippotragus niger mitochondrial genome	5	21	33400	1	34714	18142	16588
Hippotragus niger mitochondrial genome	6	21	33412	1	34738	18151	16588
Hippotragus equinus mitochondrial genome	0	26	22662	1	22773	13184	16486
Hippotragus equinus mitochondrial genome	1	25	32726	1	33917	17920	16466
Hippotragus equinus mitochondrial genome	2	22	33163	1	34537	18095	16466
Hippotragus equinus mitochondrial genome	3	22	33233	1	34763	18222	16466

Hippotragus equinus mitochondrial genome	4	23	33266	1	34802	18226	16466
Hippotragus equinus mitochondrial genome	5	23	33283	1	34840	18234	16466
Hippotragus equinus mitochondrial genome	6	23	33297	1	34862	18242	16466
Hippotragus niger CYTB	0	22	1928	1	1941	1135	1205
Hippotragus niger CYTB	1	64	7591	1	8061	3952	3482
Hippotragus niger CYTB	2	52	7054	1	7547	3669	3180
Hippotragus niger CYTB	3	52	7075	1	7580	3680	3180
Hippotragus niger CYTB	4	118	10748	1	11459	5602	4829
Hippotragus niger CYTB	5	116	10752	1	11471	5607	4829
Hippotragus niger CYTB	6	114	10753	1	11475	5609	4829
Hippotragus equinus CYTB	0	18	2210	1	2231	1292	1430
Hippotragus equinus CYTB	1	67	7591	1	8061	3952	3482
Hippotragus equinus CYTB	2	55	7054	1	7547	3669	3180
Hippotragus equinus CYTB	3	54	7075	1	7580	3680	3180

Hippotragus equinus CYTB	4	120	10748	1	11459	5602	4829
Hippotragus equinus CYTB	5	118	10752	1	11471	5607	4829
Hippotragus equinus CYTB	6	116	10753	1	11475	5609	4829
Capra hircus CYTB	0	6	338	1	341	204	239
Capra hircus CYTB	1	68	7591	1	8061	3952	3482
Capra hircus CYTB	2	56	7054	1	7547	3669	3180
Capra hircus CYTB	3	56	7075	1	7580	3680	3180
Capra hircus CYTB	4	121	10748	1	11459	5602	4829
Capra hircus CYTB	5	119	10752	1	11471	5607	4829
Capra hircus CYTB	6	117	10753	1	11475	5609	4829
Hippotragus niger ND4	0	27	1906	1	1936	1105	1954
Hippotragus niger ND4	1	159	16554	1	17368	8855	7855
Hippotragus niger ND4	2	153	13144	1	13988	7254	6515
Hippotragus niger ND4	3	152	13168	1	14022	7265	6518
Hippotragus niger ND4	4	158	19632	1	20680	10485	9300
Hippotragus niger ND4	5	158	19640	1	20702	10490	9300
Hippotragus niger ND4	6	158	19642	1	20707	10492	9300

Hippotragus equinus ND4	0	27	2950	1	2971	1674	1934
Hippotragus equinus ND4	1	147	16554	1	17368	8855	7855
Hippotragus equinus ND4	2	142	13144	1	13988	7254	6515
Hippotragus equinus ND4	3	141	13168	1	14022	7265	6518
Hippotragus equinus ND4	4	159	19632	1	20680	10485	9300
Hippotragus equinus ND4	5	159	19640	1	20702	10490	9300
Hippotragus equinus ND4	6	159	19642	1	20707	10492	9300
Capra hircus ND4	0	1	1	1	2	2	1154
Capra hircus ND4	1	1	1	1	2	2	1154
Capra hircus ND4	2	1	1	1	2	2	1154
Capra hircus ND4	3	133	12991	1	13843	7164	6519
Capra hircus ND4	4	164	19457	1	20508	10385	9301
Capra hircus ND4	5	164	19465	1	20530	10390	9297
Capra hircus ND4	6	164	19642	1	20707	10492	9300

Supplementary Table S7 Analyzed samples with GenBank accession numbers, museum collection of origin, specimen type, library type and coverage of mitochondrial genome. Horn sheaths are present unless stated otherwise.

Specimen ID	Natural history collection	Specimen type	Accession number	Analyzed material	Library type	Resulting genome	Coverage
NRM 590107	Swedish Museum of Natural History (Stockholm)	mounted skin	MW222233	skin	single-stranded	complete mitochondrial	104x
NMW ST 715	Natural History Museum Vienna, Mammal Collection	mounted skin	MW228401	skin	single-stranded	partial mitochondrial	6.2x
RMNH.MAM.20681.a	Naturalis Biodiversity Center (Leiden)	premaxilla and mandible fragments	MW228402	bone	single-stranded	partial mitochondrial	3.9x
UPSZMC 78488	Museum of Evolution (University of Uppsala)	pair of horns with traces of fur	MW222234	skin & bone	single-stranded	complete mitochondrial	17.7x
GLAHM:Z4884	The Hunterian (University of Glasgow)	cranium with horns & mandible, no horn sheaths	MW228406	bone	single-stranded	partial mitochondrial	4.1x
SAM ZM 40759	Iziko Museums of South Africa, Terrestrial Vertebrate Collection (Cape Town)	frontlet with horns	MW228407	bone	single-stranded	partial mitochondrial	2.2x
ZMA.MAM.18623	Naturalis Biodiversity Center (Leiden)	cranium with horns & mandible	MW228408	bone	single-stranded	partial mitochondrial	1.7x
BPM 2234	Bell Pettigrew Museum (University of St Andrews)	frontlet with horns	MW228403	bone	single-stranded	partial mitochondrial	13.7x
MNHN-ZM-AC-1896-100	Muséum national d'Histoire naturelle (Paris)	cranium with horns, no mandible	MW228404	bone	single-stranded	partial mitochondrial	7.3x
ZMB MAM 8859	Museum für Naturkunde, Berlin, Mammal Collection	cranium with horns, no mandible	MW228405	bone	single-stranded	partial mitochondrial	5.1x

References

1. Mohr, E. *Der Blaubock* Hippotragus leucophaeus *(Pallas, 1766) - Eine Dokumentation*. (Paul Parey, Hamburg, 1967).
2. Lindroth, P., Gustavo. *D.D. Museum Naturalium Grillium Soderforssiense*. (A. J. Nordstrom, Stockholm, 1788).
3. Kohl, F. F. Ueber neue und seltene Antilopen des k. k. naturhistorischen Hofmuseums. *Ann. K. K. Naturh. Hofmus. Wien* **1**, 75–86 (1886).
4. Husson, A. M. & Holthuis, L. B. On the type of *Antilope leucophaea* Pallas, 1766, preserved in the collection of the Rijksmuseum van Natuurlijke Historie, Leiden. *Zool. Meded.* **44**, 147–157 (1969).
5. Jentink, F. A. Aegocerus. in *Catalogue Ostéologique des Mammifères* 135 (1887).
6. Rookmaaker, L. C. Additions and revisions to the list of specimens of the extinct blue antelope (*Hippotragus leucophaeus*). *Ann. S. Afr. Mus.* **102**, 131–141 (1992).
7. Sclater, P. L. & Thomas, O. The Blue-Buck. in *The Book of Antelopes* vol. 4 5–12 (R. H. Porter, London, 1899).
8. Renshaw, G. The Blaauwbok. in *Natural History Essays* 38–58 (Sherrat & Hughes, London, 1904).
9. Temminck, C. J. *Esquisse zoologiques sur la côte de Guiné*. (E. J. Brill, Leiden, 1853).
10. Rookmaaker, L. C. *The Zoological Exploration of Southern Africa 1650-1790*. (A. A. Balkema, Rotterdam, 1989).
11. Glenn, I. The Paris bloubok (*Hippotragus leucophaeus* (Pallas, 1766) [Bovidae]) and its provenance. *Zoosystema* **42**, 77–84 (2020).
12. Harris, W. C. *Portraits of the Game and Wild Animals of Southern Africa*. (W. Pickering, London, 1840).
13. Bryden, H. A. The Blaauwbok (*Hippotragus leucophaeus*). in *Great and Small Game of Africa* (ed. Bryden, H. A.) 417–419 (Rowland Ward, Limited, London, 1899).

14. Rookmaaker, L. C., Mundy, P. A., Glenn, I. & Spary, E. *Francois Levaillant and the Birds of Africa*. (Brenthurst Press, Johannesburg, 2004).

15. Péquignot, A. The history of taxidermy: clues for preservation. *Collections* **2**, 245–255 (2006).

16. Lydekker, R. *Catalogue of the Ungulate Mammals in the British Museum (Natural History)*. vol. 3 (British Museum (Natural History), London, 1914).

17. Broom, R. The extinct blue buck of South Africa. *Nature* **164**, 1097–1098 (1949).

18. Groves, C. P. & Westwood, C. R. Skulls of the blaauwbok *Hippotragus leucophaeus*. *Z. Säugetierkd.* **60**, 314–318 (1995).

19. Klein, R. G. On the taxonomic status, distribution and ecology of the blue antelope, *Hippotragus leucophaeus* (Pallas, 1766). *Ann. S. Afr. Mus.* **65**, 99–143 (1974).

20. Ozinsky, S. Rare blue antelope horns found. *Sagittarius* **4**, 31 (1989).

21. Erdbrink, D. P. B. *Protoryx* from three localities East of Maragheh, N.W. Iran. *Proc. Kon. Ned. Akad. Wet. B* **91**, 101–159 (1988).

22. Lydekker, R. *The Game Animals of Africa*. (Rowland Ward, Limited, London, 1908).

23. Lichtenstein, H. Die Gattung Antilope. *Ges. Naturf. Freunde Berlin Mag. neuesten Entdeck. gesammten Naturk.* **6**, 147–160, 163–182 (1814).

24. Hassanin, A. *et al.* Pattern and timing of diversification of Cetartiodactyla (Mammalia, Laurasiatheria), as revealed by a comprehensive analysis of mitochondrial genomes. *C. R. Biol.* **335**, 32–50 (2012).

25. Themudo, G. E. & Campos, P. F. Phylogenetic position of the extinct blue antelope, *Hippotragus leucophaeus* (Pallas, 1766) (Bovidae: Hippotraginae), based on complete mitochondrial genomes. *Zool. J. Linn. Soc.* **182**, 225–235 (2017).

26. DeCesare, N. J. *et al.* Phylogeography of moose in western North America. *J. Mammal.* **101**, 10–23 (2020).

27. Świsłocka, M. *et al.* Phylogeny and diversity of moose (*Alces alces*, Cervidae, Mammalia) revealed by complete mitochondrial genomes. *Hystrix* **31**, 1-9 (2020).

28. Liu, H. & Jiang, G. Complete mitochondrial genome sequence of Ussurian moose, *Alces alces cameloides*. *Mitochondrial DNA Part A* **27**, 4199–4200 (2016).
29. Douglas, K. C. *et al.* Complete mitochondrial DNA sequence analysis of *Bison bison* and bison–cattle hybrids: function and phylogeny. *Mitochondrion* **11**, 166–175 (2011).
30. Achilli, A. *et al.* Mitochondrial genomes of extinct aurochs survive in domestic cattle. *Curr. Biol.* **18**, R157–R158 (2008).
31. Węcek, K. *et al.* Complex admixture preceded and followed the extinction of wisent in the wild. *Mol. Biol. Evol.* **34**, 598–612 (2016).
32. Der Sarkissian, C. *et al.* Evolutionary genomics and conservation of the endangered Przewalski's horse. *Curr. Biol.* **25**, 2577–2583 (2015).
33. Lippold, S., Matzke, N. J., Reissmann, M. & Hofreiter, M. Whole mitochondrial genome sequencing of domestic horses reveals incorporation of extensive wild horse diversity during domestication. *BMC Evol. Biol.* **11**, 328 (2011).
34. Achilli, A. *et al.* Mitochondrial genomes from modern horses reveal the major haplogroups that underwent domestication. *Proc. Natl. Acad. Sci. U.S.A.* **109**, 2449–2454 (2012).
35. Humble, E. *et al.* Chromosomal-level genome assembly of the scimitar-horned oryx insights into diversity and demography of a species extinct in the wild. *Mol. Ecol. Resour.* 1–14 (2020) doi:10.1111/1755-0998.13181.
36. Heller, R., Brüniche-Olsen, A. & Siegismund, H. R. Cape buffalo mitogenomics reveals a Holocene shift in the African human-megafauna dynamics. *Mol. Ecol.* **21**, 3947–3959 (2012).
37. Stamatakis, A. RAxML version 8: a tool for phylogenetic analysis and post-analysis of large phylogenies. *Bioinformatics* **30**, 1312–1313 (2014).
38. Meadows, J. R. S., Hiendleder, S. & Kijas, J. W. Haplogroup relationships between domestic and wild sheep resolved using a mitogenome panel. *Heredity (Edinb.)* **106**, 700–706 (2011).
39. Li, H. *et al.* The sequence alignment/map format and SAMtools. *Bioinformatics* **25**, 2078–2079 (2009).

40. Hahn, C. Assembly of Ancient Mitochondrial Genomes Without a Closely Related Reference Sequence. in *Ancient DNA - Methods and Protocols* (eds. Shapiro, B. et al.) 195–213 (Humana Press, New York, 2019).
41. Hahn, C., Bachmann, L. & Chevreux, B. Reconstructing mitochondrial genomes directly from genomic next-generation sequencing reads—a baiting and iterative mapping approach. *Nucleic Acids Res.* **41**, e129 (2013).
42. Colli, L. *et al.* Whole mitochondrial genomes unveil the impact of domestication on goat matrilineal variability. *BMC Genom.* **16**, 1115 (2015).
